# Preparation of a Sunitinib loaded microemulsion for ocular delivery and evaluation for the treatment of corneal neovascularization *in vitro* and *in vivo*

**DOI:** 10.3389/fphar.2023.1157084

**Published:** 2023-07-11

**Authors:** Jieran Shi, Jingjing Yang, Haohang Xu, Qing Luo, Jun Sun, Yali Zhang, Zhen Liang, Ningmin Zhao, Junjie Zhang

**Affiliations:** ^1^ Department of Pharmacy, Zhengzhou University People’s Hospital, Henan Provincial People’s Hospital, Zhengzhou, China; ^2^ Henan Eye Hospital, Zhengzhou University People’s Hospital, Zhengzhou, China; ^3^ First School of Clinical Medicine, Henan University of Chinese Medicine, Zhengzhou, China

**Keywords:** Sunitinib, microemulsion, ocular delivery, topical administration, corneal neovascularization, ocular pharmacokinetics

## Abstract

**Background:** Corneal neovascularization (CNV) is a pathological condition that can disrupt corneal transparency, thus harming visual acuity. However, there is no effective drug to treat CNV. Sunitinib (STB), a small-molecule multiple receptor tyrosine kinase inhibitor, was shown to have an effect on CNV. The purpose of this study was to develop an STB microemulsion (STB-ME) eye drop to inhibit CNV by topical application.

**Methods:** We successfully prepared an STB-ME by the phase inversion emulsification method, and the physicochemical properties of STB-MEs were investigated. The short-term storage stability, cytotoxicity to human corneal epithelial cells, drug release, ocular irritation, ocular pharmacokinetics and the inhibitory effect on CNV were evaluated *in vitro* and *in vivo*.

**Results:** The optimal formulation of STB-ME is composed of oleic acid, CRH 40, Transcutol P, water and sodium hyaluronate (SH). It is a uniform spherical particle with a mean droplet size of 18.74 ± 0.09 nm and a polydispersity index of 0.196 ± 0.004. In the *in vitro* drug release results, STB-ME showed sustained release and was best fitted by a Korsmeyer-Peppas model (*R* ^*2*^ = 0.9960). The results of the ocular pharmacokinetics in rabbits showed that the formulation containing SH increased the bioavailability in the cornea (2.47-fold) and conjunctiva (2.14-fold). STB-ME (0.05% and 0.1%), administered topically, suppressed alkali burn-induced CNV in mice more effectively than saline, and high-dose (0.1%) STB-ME had similar efficacy to dexamethasone (0.025%).

**Conclusion:** This study provides a promising formulation of STB-ME for the inhibition of CNV by topical administration, which has the excellent characteristics of effectiveness, sustained release and high ocular bioavailability.

## 1 Introduction

Corneal neovascularization (CNV) is a serious disease worldwide that disrupts corneal transparency, which can result in vision loss and often blindness. An ophthalmology epidemiological study showed that nearly 1.4 million people suffer from CNV, accounting for 4.14% of ocular diseases in the United States (USA) every year (Lee et al., 1998; Roshandel et al., 2018). Normally, one of the vital factors to maintain corneal optical clarity is avascularity, i.e., no capillaries or other vessels permeate into any part of the corneal structure (Nicholas and Mysore, 2021), and its avascular nature is maintained by the dynamic balance between proangiogenic and antiangiogenic factors (Barry et al., 2020). However, this balance is disrupted by pathological and physiological conditions such as inflammation, autoimmune diseases, chemical burns and corneal transplant rejection, which can lead to the growth of blood vessels. Vascular endothelial growth factor (VEGF) is an angiogenic cytokine that plays an essential role in the regeneration of existing blood vessels and the growth of new blood vessels, and previous studies have shown that VEGF-A factors are involved in approximately 50% of CNV (Aiello et al., 1995; Su et al., 2011). In addition, platelet-derived growth factors (PDGFs) have been shown to be expressed in corneal endothelial cells and epithelial cells (Kim et al., 1999), among which platelet-derived growth factor-BB (PDGF-BB) is the most abundant in the growth process of CNV.

According to previous studies, the treatments for CNV include surgery and drugs, and drug treatment is currently the main approach, including steroid hormones, immunosuppressive drugs (ISD), anti-VEGF drugs and nonsteroidal anti-inflammatory drugs (NSAIDS) (Chen et al., 2020). Although these drug treatments have shown effects on CNV, they have the disadvantage of limited efficacy and can even cause some adverse reactions, even harming ocular tissues (Bekendam et al., 2007; Feizi et al., 2017; Liu et al., 2017). Currently, many clinical and experimental studies are still being performed to develop improved drugs for the treatment of CNV. Efficacy and safety are the main characteristics to be considered in further research. Sunitinib (STB) is a multiple-target tyrosine kinase inhibitor, that has been approved by the U.S. Food and Drug Administration (FDA) for the treatment of gastrointestinal stromal tumors (GISTs) and metastatic renal cell carcinoma (RCC) (Bayyoud et al., 2014; Hao and Sadek, 2016). Due to its dual anti-VEGF and anti-PDGF effects, STB is regarded as a promising drug for the treatment of CNV (Cakmak et al., 2018). A previous study showed that STB, which inhibits both the VEGF and PDGF pathways, has a stronger inhibitory effect on CNV when administered topically than bevacizumab, which only inhibits the VEGF pathway (Perez-Santonja et al., 2010; Ko et al., 2013). The effect of R916562 on CNV in the mouse micropocket model was evaluated with STB as a positive control by oral administration (Goff et al., 2017). However, STB has the undesirable characteristics of poor hydrophilicity, slight solubility and low permeability, which limit its further application in inhibiting CNV in clinical studies. As STB free base is a BCS (Biopharmaceutics Classification System) IV compound with low permeability within the physiological pH range (Gomes Souza et al., 2023), the malate form of STB has been developed and the market is present (Sutent) that has an aqueous solubility of 25 mg/mL over the pH range of 1.2–6.8 (Sunitinib, Center for drug evaluation and research, Application number: NDA 21–938 (GIST) and NDA 21–968 (MRCC), which is significantly improved the solubility of STB in aqueous media. To enhance the corneal permeability and the bioavailability of topically administered STB, Gomes Souza and coworkers (Gomes Souza et al., 2023) recently loaded STB free base into three different types of nanocarriers polymeric nanospheres (NS), liposomes (LIP) and solid lipid nanoparticles (SLN), respectively to topically apply for treatment of CNV and SLN demonstrated the potential translation for treatment of CNV-targeting. It has been reported that STB base was encapsulated into liposomes to treat choroidal neovascularization in mouse model via the intravitreal injection and revealed inhibitory effect on established neovascularization (Tavakoli et al., 2022).

Microemulsion (ME), as one of the most promising submicron carriers for drug delivery, is spontaneously formed in the appropriate proportion of oil, surfactant, cosurfactant and water. It is not only especially suitable for drugs with poor solubility but also has physical stability and safety according to a previous study (Kumar and Sinha, 2014; Yellepeddi and Palakurthi, 2016). ME offers ocular application with additional benefits such as less frequent instillation, sustained drug action and increased ocular retention compared to conventional eye drops (Ustundag Okur et al., 2020). Reportedly, ME as a vehicle showed great adherence to the corneal surface and good permeation into the cornea, sustained drug release and ultimately improved the ocular drug bioavailability (Battaglia et al., 2016; Kalam et al., 2016; Gupta et al., 2019). In addition, ME would improve the treatment efficacy and reduce the frequency of administration to improve patient compliance (Mahran et al., 2021). Sodium hyaluronate (SH) is a thickening agent that is commonly used in eye drops, and can significantly increase the retention time of the drug on the ocular surface (Dogru et al., 2013; Chen et al., 2019). Studies have shown that SH improves corneal epithelial wound healing by promoting epithelial cell migration (Zhong et al., 2016; Asena et al., 2022). In addition, there are currently 0.3% SH eye drops on the market, such as Hialid (Santen Pharmaceutical Co., Ltd., Japan), which is sufficient to demonstrate the safety of SH. Thus, the aim of this study is to develop an ME for ocular drug delivery system of STB to overcome these shortcomings and promote the corneal permeability thus to enhance the effect of STB on CNV after topical administration.

The purpose of this study was to develop an ocular delivery system of STB-ME for topical application and optimize the formulation by the central composite design response surface method (CCD-RSM). Then, the physical and chemical properties of STB-ME were characterized, and the *in vitro* drug release kinetics were investigated. Human corneal epithelial cell (HCEC) cytotoxicity was evaluated *in vitro*, and ocular irritation in rabbits was evaluated *in vivo*. The ocular pharmacokinetics after one single dose of topical administration was investigated in rabbits; moreover, the pharmacodynamics of STB-ME were evaluated in the treatment of alkali burn-induced CNV in mice.

## 2 Materials and methods

### 2.1 Materials

STB was acquired from Macklin (Shanghai, China). SH with a molecular weight (MW) of 1,460 kDa was acquired from Bloomage Freda Biopharm Co., Ltd. (Jinan, China). Oleic acid (OA) was purchased from TCI (Shanghai, China). Transcutol P (TSP) was obtained from Gattefossé (Saint-Priest, France). CremophorRH40 (CRH 40) was acquired from BASF SE (Ludwigshafen, Germany).

### 2.2 Animals

New Zealand white rabbits weighing 2.0–2.5 kg and BALB/c mice weighing 18–22 g (6–8 weeks of age) were purchased from Huaxing Experimental Animal Breeding Co. (Zhengzhou, China). All animals were freely supplied with healthy food and water and were housed in a room at 22.0°C ± 3°C. In addition, there was a 12-h on 12-h off lighting schedule to ensure normal and regular life for the animals. All animal experiments conformed to the statements of ARVO (Association for Research in Vision and Ophthalmology). All animal procedures were approved by the Experimental Animal Ethics Committee of Henan Institute of Ophthalmology.

### 2.3 Assay of STB by high-performance liquid chromatography

STB was quantitatively assayed by high-performance liquid chromatography (HPLC) according to the previous literature (Blanchet et al., 2009; Iqbal et al., 2014), and the flow rate and column were adjusted to be more suitable for the detection of STB. Briefly, HPLC (Waters 2,695 liquid chromatography system) (Milford, MA, United States) was used in the formulation of CCD-RSM, a short-term storage stability study, an *in vitro* drug release study and an *in vivo* ocular pharmacokinetics study. An X-Bridge C18 column (3.5 μm, 3.0 × 150 mm) was used with a column temperature of 40°C. The mobile phase consisted of ammonium acetate buffer (0.02 mol/L) and acetonitrile (62: 38, V/V) at a flow rate of 0.4 mL/min. Twenty microliters of sample was injected, and the ultraviolet (UV) detection wavelength was set at 430 nm. The limit of quantitation (LOQ) of STB for HPLC was 25 ng/mL. The concentration of STB was calculated from the standard curve. The standard curve referred to the curve containing different concentrations of STB diluted with methanol. The quantitative analysis methods of STB followed the ICH guidelines, details of which are reported in the Supplementary Section S2.

### 2.4 Preparation of STB-ME

The formulation was optimized using the pseudo ternary phase diagrams method and CCD-RSM (Supplementary Section S1). According to the results, the formulation was composed of OA (0.20 g), CRH 40 (1.23 g), TSP (0.57 g) and STB (0.02 g) and prepared by the phase inversion emulsification method. The oil, surfactant and cosurfactant were mixed, the drug was added and thoroughly dissolved by gently stirring with a smart magnetic stirrer (37°C, 600 rpm), and then purified water at the same temperature was added dropwise and gently stirred until the solution was uniform and transparent. Then, 10 mL of SH (0.6%) solution was added to the STB-ME, and the total volume of the final mixture solution was 20 mL.

### 2.5 Characterization of STB-ME

#### 2.5.1 Determination of droplet size (DS), polydispersity index (PDI) and zeta potential (ZP)

The mean DS, PDI and ZP of the STB-ME formulations were measured by dynamic light scattering (DLS) (Zetasizer, NanoZS90, Malvern Instruments, Worcestershire, United Kingdom), with light scattering set at 25°C at an angle of 90 (Li et al., 2015). All STB-ME samples were diluted at a ratio of 1: 20 with purified water and filtered with a 0.22 μm filter.

#### 2.5.2 Assessments of encapsulation efficiency (EE) and drug loading (DL)

The STB-ME samples were placed in an ultrafiltration tube with an MW cut off of 10 kDa (Amicon^®^ Ultra-4, Merck Millipore Ltd. Ireland) and centrifuged (4,000 rpm, 4°C) by a low-temperature centrifuge for 10 min. The filtrate was obtained from the bottom of the ultrafiltration tube by centrifugation, while the STB content of STB-ME was analyzed without centrifugation. The filtrates and the STB-ME samples were diluted with methanol in different proportions, and then the STB content was analyzed by HPLC (Section 2.3). The drug loading (DL) and encapsulation efficiency (EE) were calculated by the following two equations (Vieira et al., 2020; Ma et al., 2022):

$$DL\;\left(\%\right)=\frac{W_{t}-W_{f}}{W_{m}}\times 100\%$$ (1)

$$EE\;\left(\%\right)=\frac{W_{t}-W_{f}}{W_{t}}\times 100\%$$ (2)

where $W_{t}$ is the mass of total STB in STB-ME, $W_{f}$ is the mass of free STB after centrifugation, and $W_{m}$ is the mass of STB and lipid.

#### 2.5.3 Measurements of pH and osmolality

To ensure that the STB-ME formulation met the requirements for eye drops, pH and osmolality needed to be tested. The pH value of the STB-ME formulation was measured by a pH meter (PHS-3C, Shanghai Precision & Scientific Instrument Co., Ltd., Shanghai, China), and the osmolality value was measured by a freezing point osmometer (STY-1A, Tianda Tianfa Technology Corp., Ltd., Tianjin, China). All STB-ME samples were tested in triplicate.

#### 2.5.4 Morphological observation of STB-ME

Transmission electron microscopy (TEM) (Joel JEM 1230, Tokyo, Japan) was used to observe the morphological characteristics and DS of the optimal formulation of STB-ME. Phosphotungstic acid (2%) was placed on a carbon-coated copper grid, which was topped with a 50-fold dilution of STB-ME. After the copper grid was air-dried at room temperature, the sample morphology was observed by TEM.

#### 2.5.5 Assessments of Fourier transform infrared (FTIR) spectroscopy

FTIR was one of the methods for determining the sample structure. An FTIR spectrometer (Alpha II, Bruker, Germany) was set at wavenumbers of 500–4,000 cm^−1^ with a resolution of 4 cm^−1^ to obtain the pure STB, blank-ME, STB-ME of the optimized formulation and a physical mixture between STB and blank-ME, and their FTIR spectra were compared (Li et al., 2009).

### 2.6 Short-term storage stability

The stability was an important factor in evaluating the properties of the STB-ME formulation. The optimal STB-ME formulation was sealed and stored at different temperatures (4, 25°C and 40°C) for 3 months, during which the values of DS, PDI, pH and EE were determined at 0, 1, 2 and 3 months, respectively (Kalam et al., 2010). Samples stored at each temperature were analyzed in triplicate at different time points.

### 2.7 *In vitro* HCEC cytotoxicity study

The safety of STB-ME *in vitro* was evaluated by the viability of HCECs via a Cell Counting Kit-8 (CCK-8) assay. HCEC in medium (5% serum and 1% dual antibody, 100 μL) was seeded into 96-well plates at a density of 1 × 10^4^ cells per well and incubated in a carbon dioxide incubator (5% CO_2_, 37°C) for 24 h. Then, 100 μL of 10 and 20 μg/mL STB-ME and blank-ME were used as the test groups; the medium was used as the control group; and each group was cultured for 15 min, 1, 2 and 4 h, respectively. After each sample was aspirated, CCK-8 solution containing medium (10%, 100 μL) was added and cultured for 3 h. The absorbance of each well was measured with a microplate reader (Multiskan FC, Thermo Scientific, Shanghai, China) at a wavelength of 450 nm, and the following equation was used to calculate cell viability (Yin et al., 2021):

$$V=\frac{{OD}_{t}-{OD}_{b}}{{OD}_{c}-{OD}_{b}}\times 100\%$$ (3)

where V is the cell viability, ${OD}_{t}$ is the absorption of the test group, ${OD}_{b}$ is the absorption of the blank group and ${OD}_{c}$ is the absorption of the control group.

### 2.8 *In vitro* drug release

The *in vitro* drug release of STB from STB-ME was studied using the dialysis bag method, which was performed in a constant temperature oscillator (100 rpm, 37°C). STB-ME (0.1%, 1 mL) was added to a dialysis bag (MW: 3500 D), and the release medium (250 mL, pH = 7.4) was simulated tear fluid (STF) containing 0.1% Tween 80 (Hagerstrom et al., 2000). The release solution (1 mL) was drawn at 0.25, 0.5, 1, 2, 4, 6, 8, 10, 12, 24, 36, 48, 72 and 96 h, and equal amounts of blank release solution were added (Gunday Tureli et al., 2017; Akram et al., 2019; Liang et al., 2021). The content of STB in the release solution was determined by HPLC (Section 2.3) and calculated by the Eq. 4, in which all samples were made in triplicate. The release amount of STB-ME was fitted by zero-order, first-order, Higuchi and Korsmeyer-Peppas equation models, and the most appropriate fitting model was selected by comparing the parameters of the regression coefficient squared (*R* ^*2*^) and slope (*K*). Origin software (Version 2019.b) was used to draw the release curve and fit the equation of STB-ME.

$$Q\left(\%\right)=\frac{V_{0}C_{n}+V\sum_{i=1}^{i=n-1}C_{i}}{W_{0}}\times 100\%$$ (4)

where $V_{0}$ is the total volume of released medium (250 mL), V is the volume sampled (1 mL), $C_{n}$ is the drug concentration at the time N time, $C_{i}$ is the drug concentration at Ti, and $W_{0}$ is the total mass of STB (1 mg) in the dialysis bag.

### 2.9 Ocular irritation test in rabbits

The rabbit ocular irritation test of STB-ME was performed using the Draize test on 6 New Zealand white rabbits. Before the experiment, the eyes of each rabbit were examined with a slit lamp (SLM-8E, Chongqing Kanghua Ruiming Science Technology Co., Ltd., Chongqing, China) to confirm that the ocular tissues (cornea, conjunctiva and iris) were normal and free from inflammation and lesions. STB-ME (0.1%, 100 μL) was instilled into the conjunctiva sac of the right eye as the test group, and the same dose of saline was instilled into the conjunctiva sac of the left eye as the control group. After a single topical administration, the conjunctival sac of the rabbit was gently closed for 15 s to prevent leakage of the applied solution. At different time points (0, 1, 2, 4, 24, 48 and 72 h) after administration, each eye was photographed with a slit lamp under both visible light and cobalt blue light, and irritation was scored according to Draize rules. The mean value of the irritation score was calculated, and a score ranging from 0 to 3 was considered non-irritating. After 72 h, the rabbits were euthanized with an overdose of sodium pentobarbital solution (4%, w/v) via ear intravenous injection, and eyeballs were made into histological sections and stained with hematoxylin and eosin (H&E) to observe the pathological status under a microscope (Nikon 80i, Nikon Corporation, Tokyo, Japan) (Liu et al., 2012; Li et al., 2021; Yang et al., 2022).

### 2.10 Ocular pharmacokinetics in rabbit eyes study

#### 2.10.1 Animal treatments

Forty-eight healthy New Zealand white rabbits without eye disease were randomly divided into test and control groups. Both eyes in the test group received a single dose of 50 μL of STB-ME containing 0.3% SH, and both eyes in the control group were given 50 μL of STB-ME without SH. The twenty-four rabbits in each group were allotted to 8 time points (0.25, 0.5, 1, 2, 4, 6, 8 and 12 h). After topical administration in the conjunctival sac, the eyes were gently closed for 15 s. The rabbits were euthanized with an overdose of sodium pentobarbital solution (4%, w/v) via ear intravenous injection, and the conjunctiva and cornea were harvested with forceps and surgical scissors. The harvested ocular tissues were rinsed with saline, blotted with filter paper, and weighed immediately. All samples were sealed and stored at −80°C until the drug needed to be assayed (Chandasana et al., 2014; Huang et al., 2021).

#### 2.10.2 Analysis of STB in ocular biological samples

A validated method according to previously reported (Li et al., 2018) was modified to extract STB from rabbit cornea and conjunctiva. Briefly, the conjunctiva and cornea of a certain mass were cut and soaked in methanol (0.4 mL), vortexed for 1 min and then stored at 4°C for 24 h. All samples mixed with methanol were centrifuged (12,000 rpm, 10 min), and then the supernatant was drawn and determined by the same HPLC method as described in Section 2.3. The bioanalytical methods for the quantitative analysis of STB in the conjunctival and corneal samples were validated for specificity, linearity, recovery, precision, accuracy and stability, and successfully applied to assay the levels of STB in the tissue samples for the pharmacokinetics study (Supplementary Section S2).

### 2.11 Study on anti-CNV effects in mice

#### 2.11.1 Model establishment of CNV

After general anesthesia with sodium pentobarbital and topical anesthesia with proparacaine hydrochloride, mice were subjected to alkali burns. The NaOH solution (2 μL, 1 mol/L) was dropped onto circular filter paper (diameter of 2 mm), which was attached to the center of the right eye for 20 s and removed. The conjunctival sac was quickly washed with saline solution (20 mL, 0.9%), and no operation was performed on the left eye. The mice were divided into five groups (saline solution, 0.025% STB-ME, 0.05% STB-ME, 0.1% STB-ME, 0.025% dexamethasone (DEX)), with 16 mice in each group. Each group was given 5 μL of the drug twice a day for 7 days (Sun et al., 2019).

#### 2.11.2 Observation and measurement of CNV

The growth of CNV was observed and photographed under a slit lamp on days 1, 3 and 7 for comparative study, and the corneal burned area was photographed by fluorescein sodium staining on day 0 to verify the consistency of the model. After 7 days, 3 mice from each group were randomly anesthetized by intraperitoneal injection of pentobarbital sodium solution (0.3 mL, 1%). The hearts of the mice were perfused with heparinized saline and then hematoxylin, and the right cornea was removed and sectioned to observe CNV. In addition, the CNV area was calculated using the following equation (Huang et al., 2015):

$$S=\frac{C}{12}\times 3.1416\times \left[r^{2}-{\left(r-L\right)}^{2}\right]$$ (5)

where C is the number of clock hours of CNV involved, r is the radius of the cornea and L is the length of the vessel from the limbus.

#### 2.11.3 Histopathological examination

After the mice were sacrificed on day 7, three mice from each group were randomly selected for the whole eyeball. After the corneas were trimmed, they were fixed with 4% paraformaldehyde for 48 h and embedded in wax-tissue. Sagittal planes (5 μm) were stained with H&E, and images were captured using a microscope (Mohammadpour et al., 2015).

#### 2.11.4 Enzyme-linked immunosorbent assay (ELISA)

On days 3 and 7 after alkali burns, 5 mice were randomly selected and sacrificed by injecting an overdose of 1% sodium pentobarbital solution. Then, corneal tissue samples were dissected, weighed and stored at −80°C. Before testing, corneal samples were placed at 4°C for half an hour and cut into pieces. Afterward, the samples were immersed in 30 μL of radio immunoprecipitation assay (RIPA) lysis buffer from ELISA Kits (Solarbio^®^ Life Sciences Company, Beijing, China) in an ice bath for 1.5 h and centrifuged for 5 min (4°C, 12,000 rpm). The supernatant was extracted, the total protein content was determined by a BCA Protein Assay Kit (Solarbio^®^ Life Sciences Company, Beijing, China), and the contents of VEGF-A and PDGF-BB were detected by a Mouse ELISA Kit (Elabscience^®^ Biotechnology Co., Ltd., Wuhan, China). The absorbance of each well on a Micro ELISA Plate Reader was tested by a microplate reader (Multiskan FC, Thermo Scientific, Shanghai, China) at 450 nm (Hara et al., 2010; Irani et al., 2017).

### 2.12 Statistical data analysis

In all experiments, each sample was made in triplicate and the mean and standard deviation (SD) were calculated, which were expressed as the mean ± SD. The figures of results were plotted by Origin software (Version 2019.b), including pseudo ternary phase diagrams, line graphs and bar charts. SPSS software (SPSS 21.0 Version) was used to analyze the difference in data, an independent sample t-test was used to compare two groups of data in pharmacokinetics, and ANOVA followed by Tukey as a *post hoc* test was used to compare the differences between three or more groups of data in the anti-CNV test. The difference was represented by the value *p*, and *p* < 0.05 indicated a significant difference. Pharmacokinetics parameters were calculated using DAS 2.1.1 software (Anhui Provincial Center for Drug Clinical Evaluation, Wuhu, China).

## 3 Results

### 3.1 Characterization of STB-ME

**FIGURE 1:**
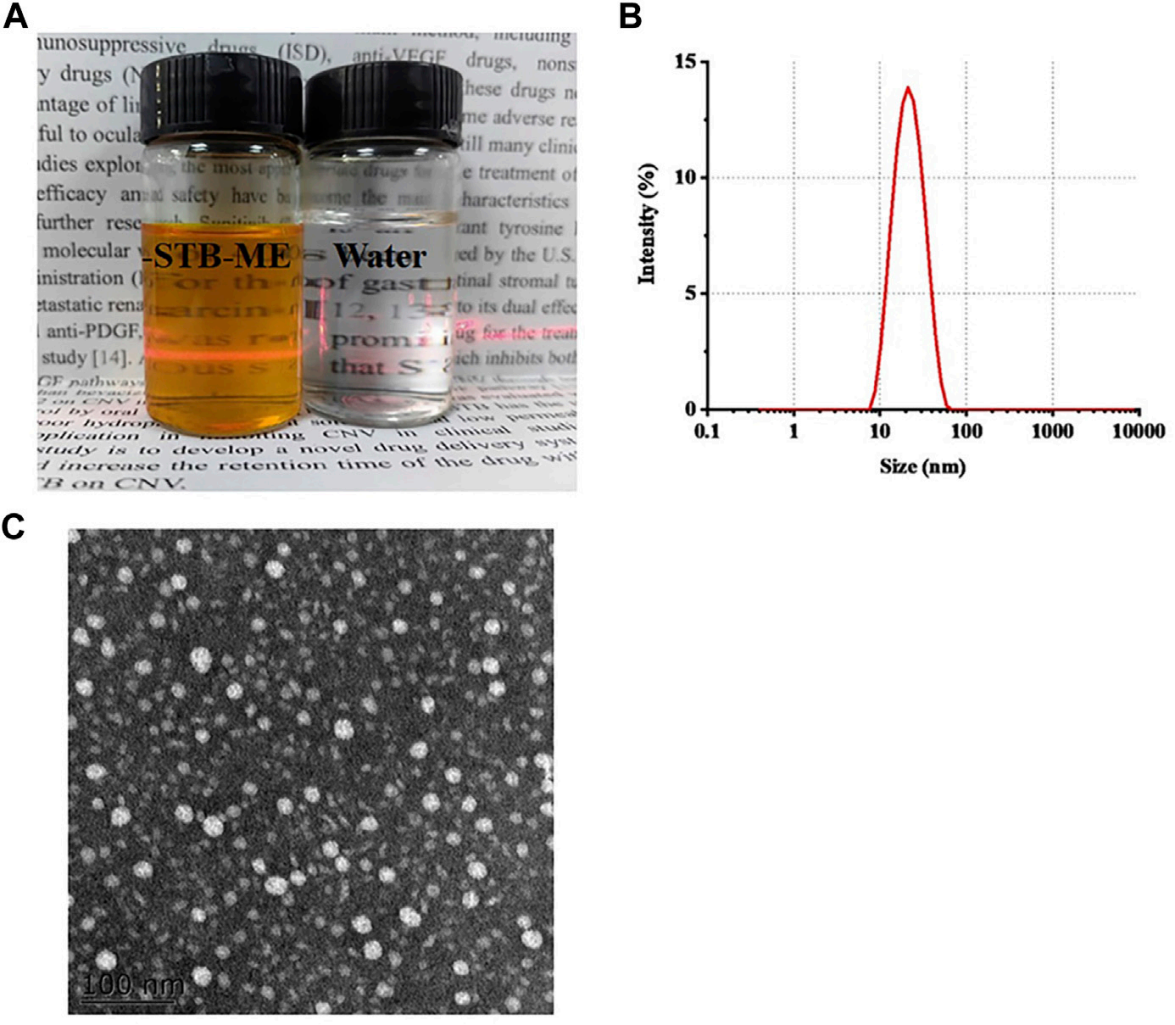
The appearance **(A)** (orange: STB-ME; white: water), the DS distribution **(B)** and the TEM image **(C)** of STB-ME.

The appearance of the optimal STB-ME was an orange transparent solution with the Tyndall effect (Figure 1A), and the characteristics were shown in Table 1, including pH, osmolality, DS, PDI, DL and EE. Both the pH (5.86 ± 0.02) and osmolality (292 ± 0.58 mOsm/kg) indicated that the STB-ME met the requirements for eye drops. The DS (18.74 ± 0.09 nm) and PDI (0.196 ± 0.004) were small, showing the uniform distribution of the particles without aggregation (Figure 1B), which was consistent with the results of the TEM image (Figure 1C). In addition, the DL (8.73% ± 0.04%) and EE (99.27% ± 0.02%) indicated good performance of STB-ME.

**TABLE 1:** Characteristics of optimal STB-ME (mean ± SD, *n* = 3).

pH	DS (nm)	PDI	ZP (mV)	Os (mOsm/kg)	DL (%)	EE (%)
5.86 ± 0.02	18.74 ± 0.09	0.196 ± 0.004	−19.63 ± 1.65	292 ± 0.58	8.73 ± 0.04	99.27 ± 0.02

Abbreviations: DS, droplet size; PDI, polydispersity index; ZP, zeta potential; Os, osmolality; DL, drug loading; EE, encapsulation efficiency.

**FIGURE 2:**
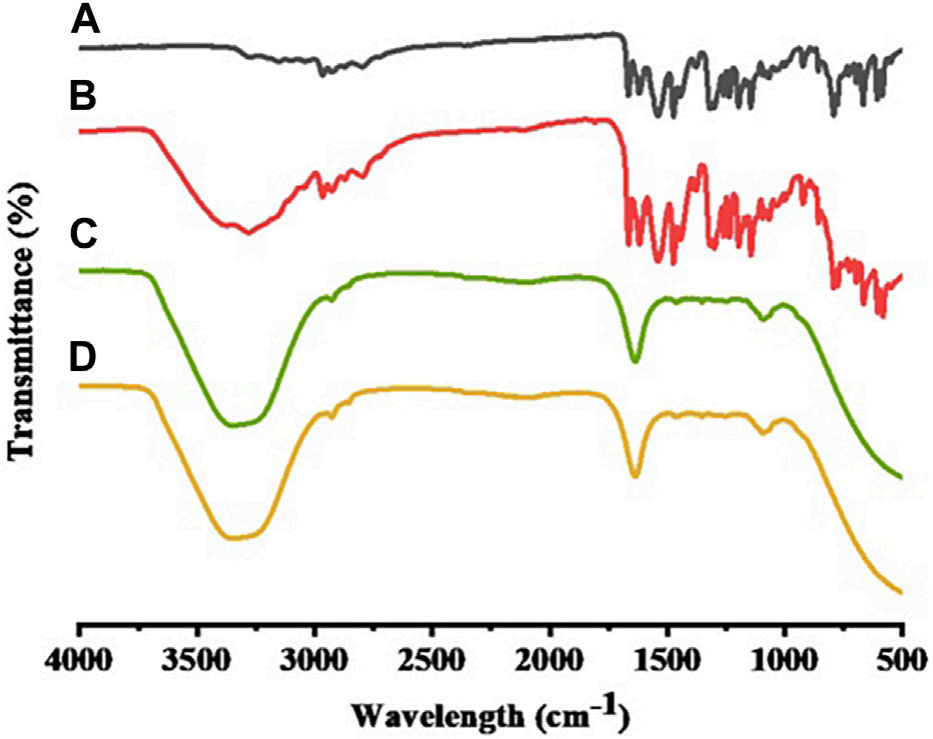
FTIR spectra of pure STB **(A)**, a physical mixture between STB and blank-ME **(B)**, STB-ME **(C)** and blank-ME **(D)**.

FTIR determined the formation of STB-ME by identifying changes in the position, frequency and character of characteristic peaks of STB. The FTIR spectra of STB, blank-ME, STB-ME and a physical mixture between STB and blank-ME samples were shown in Figure 2. The FTIR spectrum showed that the main characteristic peaks of pure STB (Figure 2A) were at 1739 cm^−1^ for C=C–F, 1,667 cm^−1^ for C=O stretching of the amide, 1,474 cm^−1^ for C=C stretching of the aromatic ring and 1,319 cm^−1^ for C–N bending, which was in line with previous literature (Chegini et al., 2019). All of the abovementioned characteristic peaks of pure STB were displayed in the physical mixture between STB and the blank-ME spectrum (Figure 2B), but were absent in the STB-ME spectrum (Figure 2C). In addition, the characteristic peaks of STB-ME were the same as those of blank-ME (Figure 2D) in the FTIR spectrum, indicating that the STB in STB-ME was completely dissolved in oil phase (OA).

### 3.2 Short-term storage stability

**FIGURE 3:**
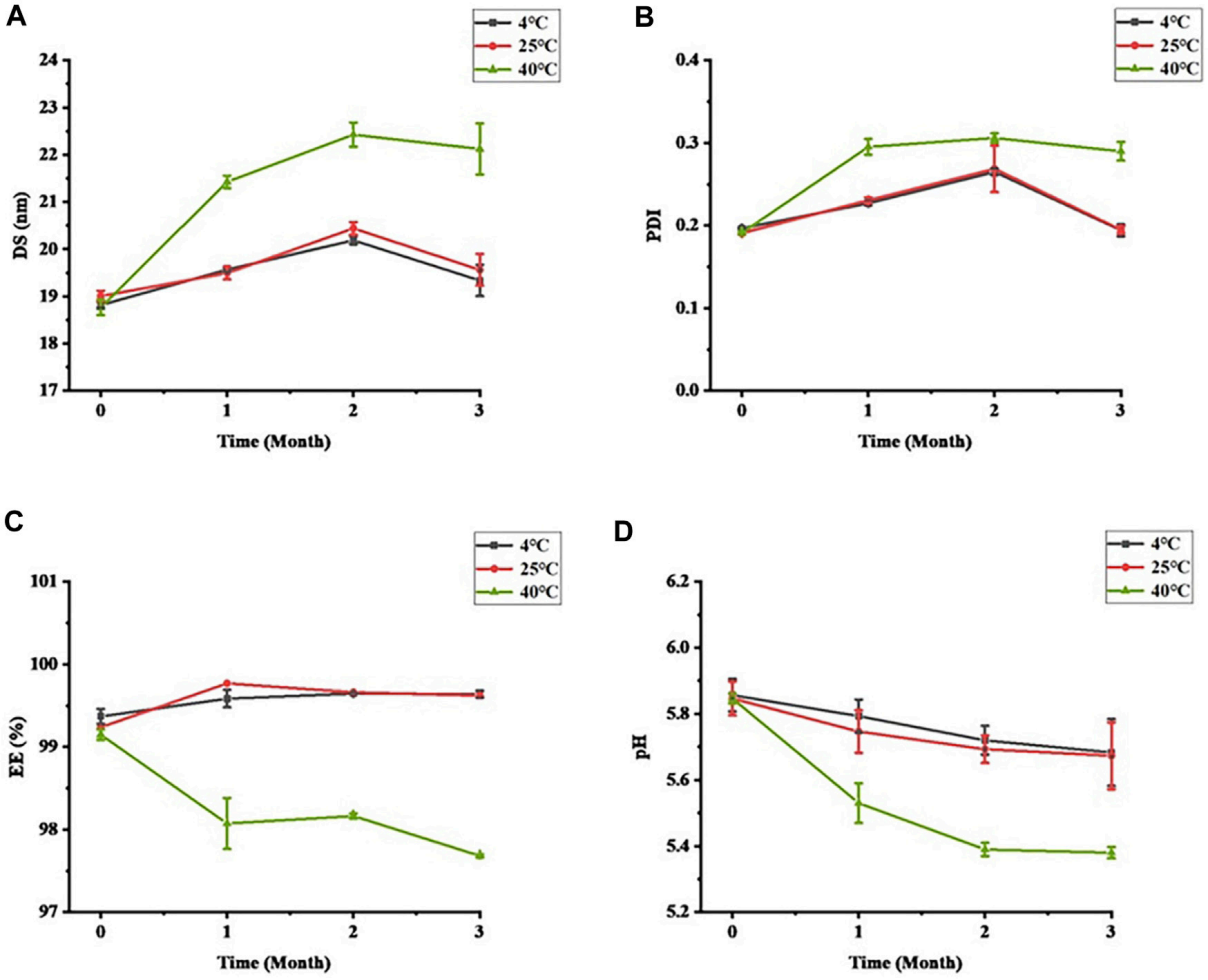
The short-term stability of STB-ME. **(A)** DS, **(B)** PDI, **(C)** EE, **(D)** pH (mean ± SD, *n* = 3).

Stability is an important property for evaluating formulations. The stability of STB-ME tested at different temperatures (4, 25°C and 40°C) is shown in Figure 3. The color and clarity of STB-ME at 4°C and 25°C did not change, and there was no significant difference in the characterization (DS, PDI, pH and EE), showing excellent physical stability. However, the viscosity decreased and the fluidity increased at 40°C because SH was unstable at high temperature. At 40°C, the DS and PDI of STB-ME had great differences (*p* < 0.05), the DS changed from 18.77 ± 0.17 nm at 0 months to 22.12 ± 0.54 nm at 3 months, and the PDI changed from 0.191 ± 0.002 at 0 months to 0.290 ± 0.011 at 3 months. In addition, pH decreased from 5.85 ± 0.02 to 5.38 ± 0.02, and EE decreased from 99.15% ± 0.06% to 97.68% ± 0.02%, both of which were significantly different (*p* < 0.05). Therefore, STB-ME should be stored at 4°C and 25°C but cannot be stored at higher temperatures.

### 3.3 *In vitro* human corneal epithelial cell (HCEC) cytotoxicity

**FIGURE 4:**
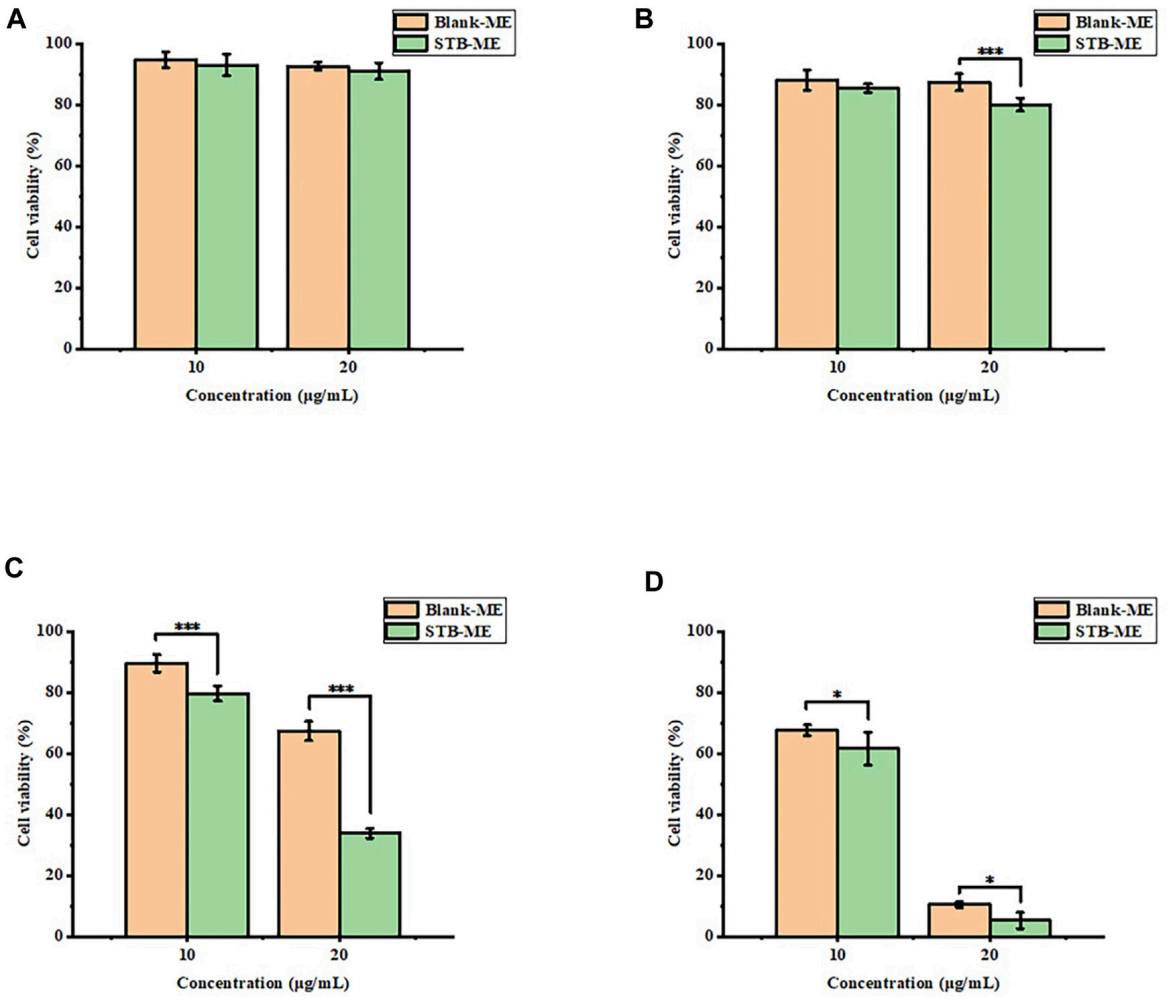
The cell viability of STB-ME at different concentrations and different times. **(A)** 0.25 h, **(B)** 1 h, **(C)** 2 h, **(D)** 4 h (*p < 0.05, ***p < 0.001; mean ± SD, *n* = 6).

A CCK-8 assay was used to detect the cell viability of STB-ME at different concentrations and different period, as shown in Figure 4. The viability of HCECs treated with 20 μg/mL STB-ME was more than 80% within 1 h of continuous exposure, indicating that it had no obvious toxicity, but the cell viability gradually decreased from 2 h of continuous exposure. STB-ME at 10 μg/mL showed no cytotoxicity within 2 h of continuous exposure, and the cell activity began to decline after 4 h. The results of the *in vitro* cytotoxicity assay showed that the cell activity was positively correlated with the concentration and administration time of STB-ME. However, the concentrations of 10 and 20 μg/mL corneal epithelial cells could not be reached for a long time because of tear flushing and enzyme metabolism in the ocular tissues.

### 3.4 *In vitro* drug release

**FIGURE 5:**
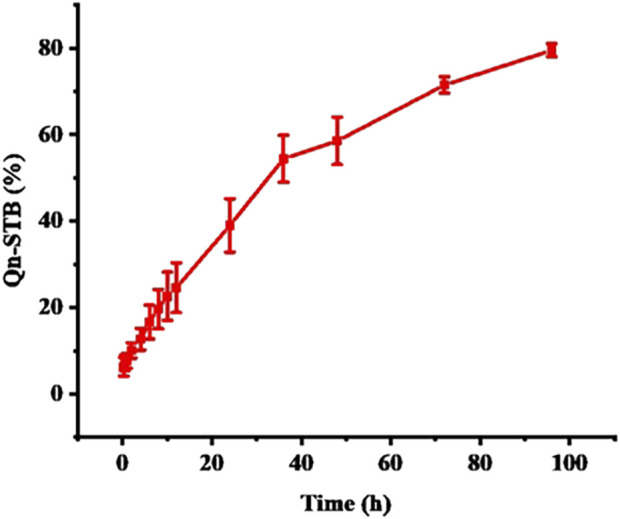
The *in vitro* drug release curve of STB-ME (mean ± SD, *n* = 3).

*In vitro* drug release simulates the process of drug release from the delivery system and provides an indispensable reference for *in vivo* studies. The cumulative amount of STB released (Figure 5) reached approximately 75% at 96 h and was sustained. Four different kinetic models were used to analyze the release mechanism of STB-ME by comparing the relevant parameters and regression coefficients. According to the fitting results of the mathematical models of the drug release curve (Table 2), STB-ME was more suitable for the Korsmeyer-Peppas model (*R* ^*2*^ = 0.9960).

**TABLE 2:** Relevant parameters and regression coefficients of different mathematical models fitting for STB release *in vitro*.

Formulation	Zero order		First order		Higuchi		Korsmeyer-Peppas		
	K (h)	*R* ^*2*^	K (h)	*R* ^*2*^	K (h^1/2^)	*R* ^*2*^	K (h)	*n*	*R* ^*2*^
STB-ME	0.7808	0.9775	0.0349	0.9806	8.2381	0.9956	7.5263	0.5197	0.9960

### 3.5 Ocular irritation test in rabbits

**FIGURE 6:**
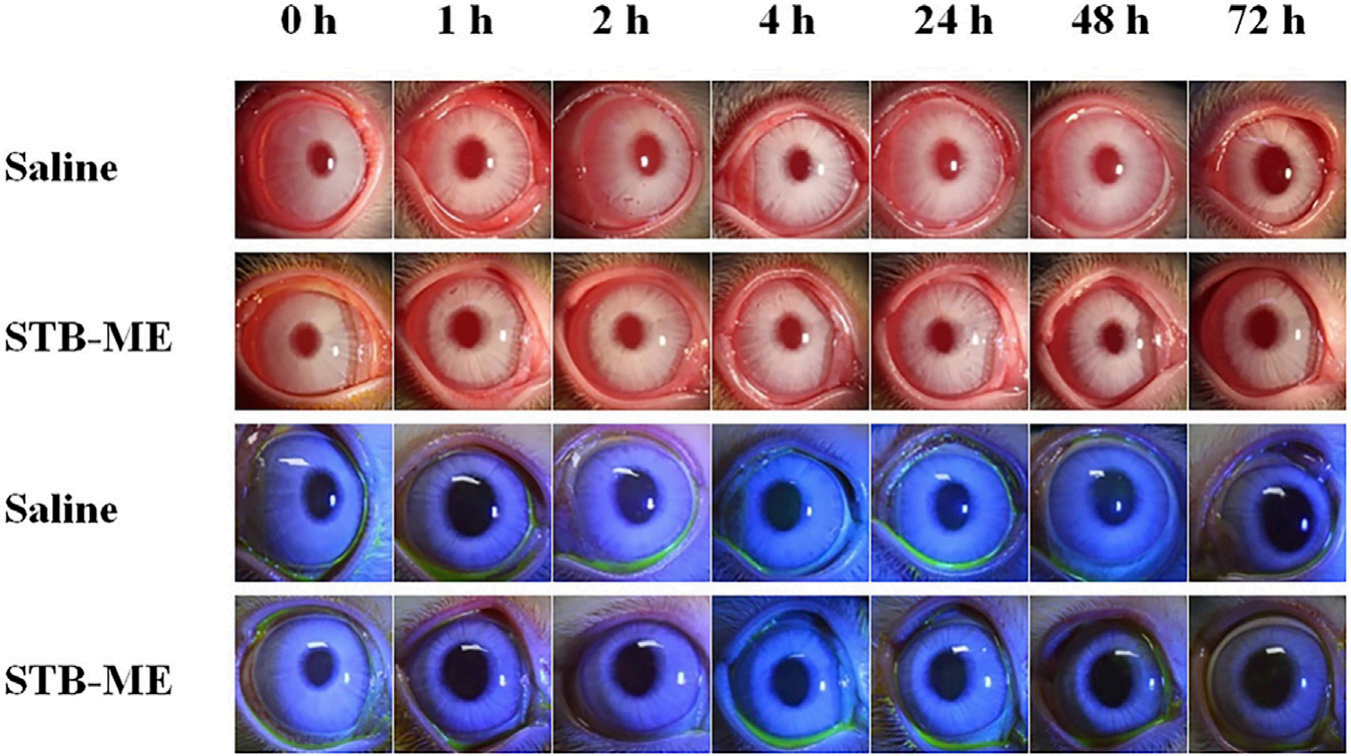
Visible light and cobalt blue light photographs after a single topical administration at different times.

**FIGURE 7:**
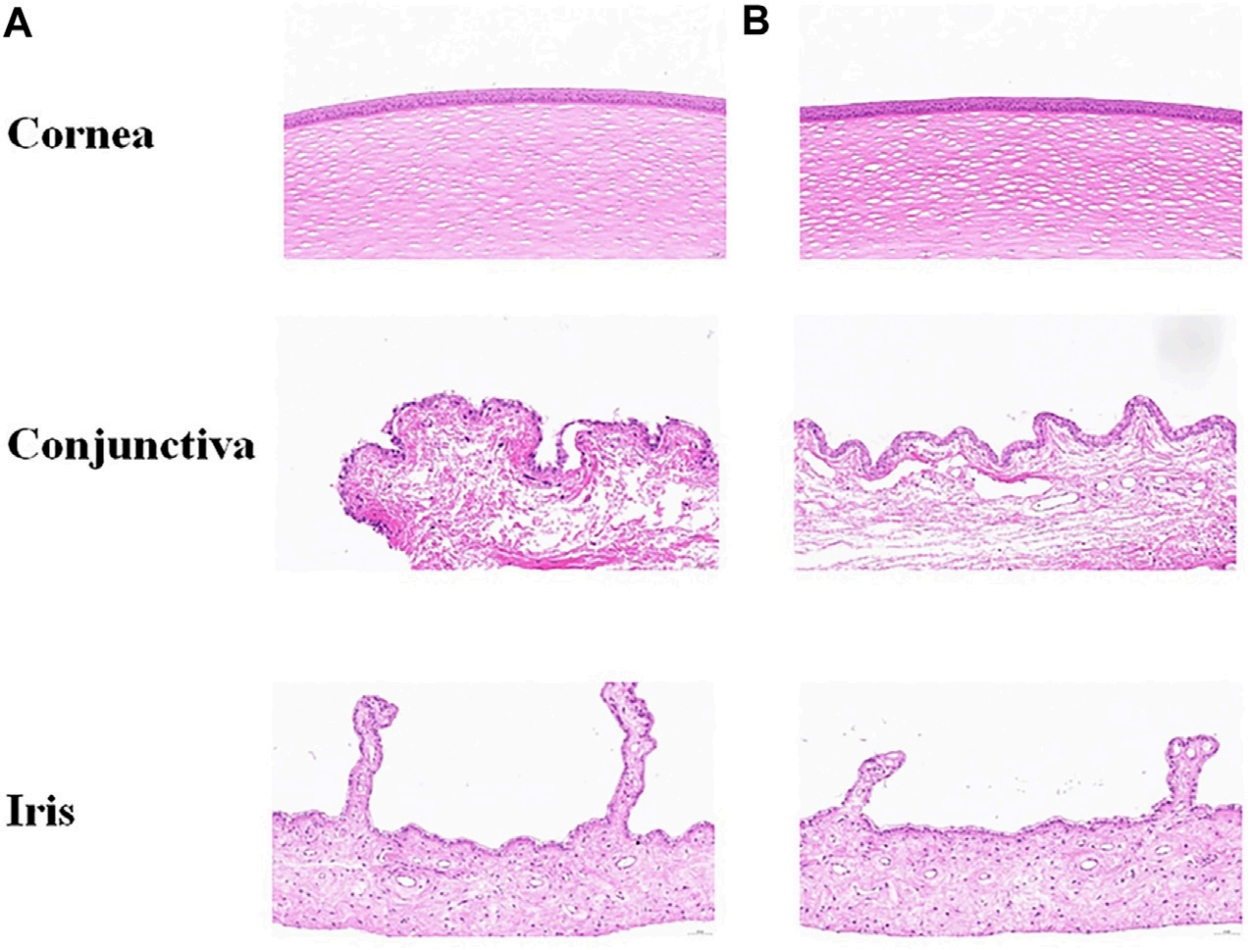
Images of ocular tissues after a single topical administration at 72 h. **(A)** Left eye: saline; **(B)** right eye: STB-ME.

Visible light and cobalt blue light (fluorescein sodium staining) photographs were taken at different time points after a single topical administration. The left eye was administered saline as the control group, and the right eye was administered STB-ME as the test group (Figure 6). The scanning images of the ocular section (cornea, conjunctiva and iris) after administration are shown in Figure 7. There was no edema or secretion in the eyes, and there were no abnormalities in the cornea, iris or conjunctiva, showing that the ocular irritation score was 0. In addition, there were no obvious side effects on the ocular structure and integrity, indicating no irritation or corrosion after STB-ME administration.

### 3.6 Ocular pharmacokinetics in rabbit eyes

**FIGURE 8:**
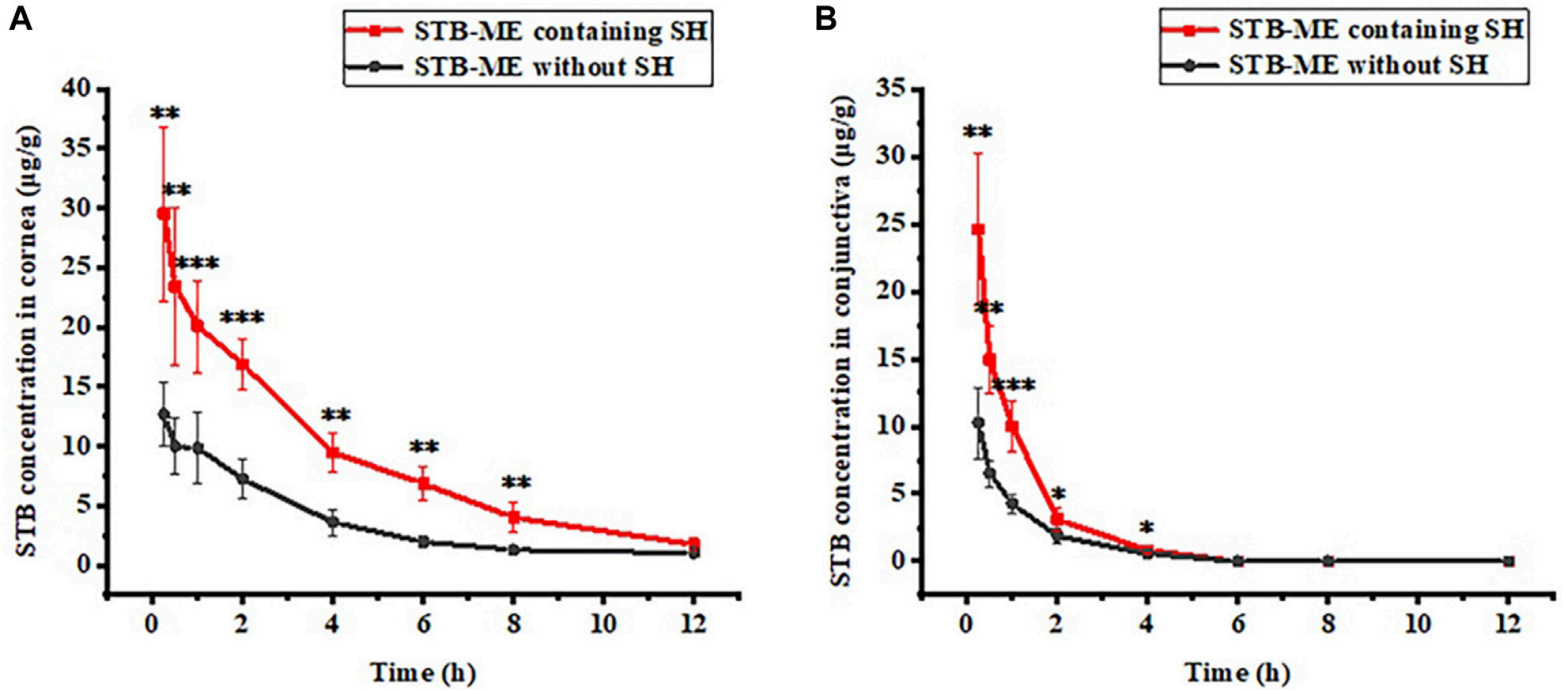
The pharmacokinetic curves of **(A)** cornea and **(B)** conjunctiva after a single topical administration. (The IC_50_ of STB inhibiting VEGF was 80 nmol/L (32 ng/mL), *p < 0.05, **p < 0.01, ***p < 0.001; mean ± SD, *n* = 6).

The STB concentrations in the cornea and conjunctiva at different times after one single-dose topical administration were shown in Figure 8, indicating that 0.3% SH significantly increased the concentration of STB in the ocular tissues. After the administration of STB-ME containing SH, the STB concentration in the cornea (Figure 8A) before 8 h and in the conjunctiva (Figure 8B) before 4 h increased significantly (*p* < 0.05) at corresponding time points. The AUC_(0–12 h)_ of the pharmacokinetic results (Table 3) showed that STB-ME was 2.47–and 2.14 -fold higher than STB-ME without 0.3% SH in the cornea and conjunctiva. In addition, compared with STB-ME without SH, the C_max_ values increased from 12.67 ± 2.68 μg/g to 29.53 ± 7.33 μg/g in the cornea and from 10.27 ± 2.63 μg/g to 24.56 ± 5.62 μg/g in the conjunctiva in STB-ME with SH. Furthermore, the levels of STB in the cornea and conjunctiva in both groups reached a maximum at 0.25 h (T_max_) and then decreased. Sunitinib inhibited VEGF-stimulated endothelial cell proliferation with an IC_50_ value of 80 nmol/L (32 ng/mL) (Zhang et al., 2018), which is far lower than the C_max_ in the cornea and conjunctiva which was assayed in this experiment. In this experiment, the levels of STB in the cornea within 12 h and in the conjunctiva within 4 h were higher than the IC_50_ value, indicating that STB could inhibit CNV during these periods.

**TABLE 3:** The pharmacokinetic parameters of ocular tissues after one single dose topical administration.

Tissue	Pharmacokinetic parameters	STB-ME containing SH	STB-ME without SH
Cornea	T_1/2_ (h)	3.07	2.46
	T_max_ (h)	0.25	0.25
	AUC _(0–12h)_ (μg/g *h)	104.80	42.36
	C_max_ (μg/g)	29.53 ± 7.33	12.67 ± 2.68
Conjunctiva	T_1/2_ (h)	0.83	0.84
	T_max_ (h)	0.25	0.25
	AUC _(0–12h)_ (μg/g* h)	24.78	11.59
	C_max_ (μg/g)	24.56 ± 5.62	10.27 ± 2.63

### 3.7 Study on anti-CNV

#### 3.7.1 Observation and measurement of CNV

**FIGURE 9:**
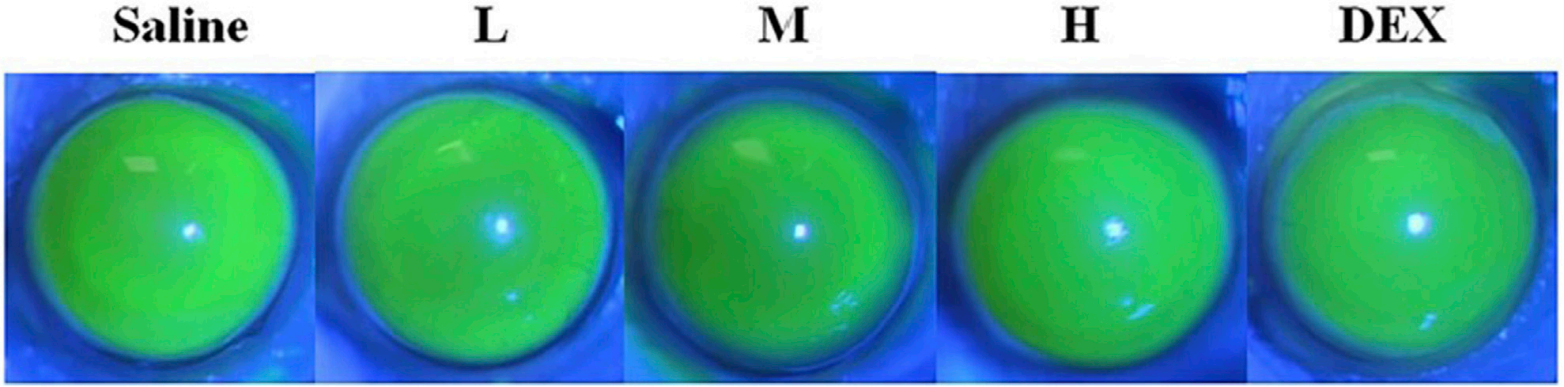
Fluorescein sodium images after modeling on day 0. (L: 0.025% STB-ME, M: 0.05% STB-ME, H: 0.1% STB-ME).

**FIGURE 10:**
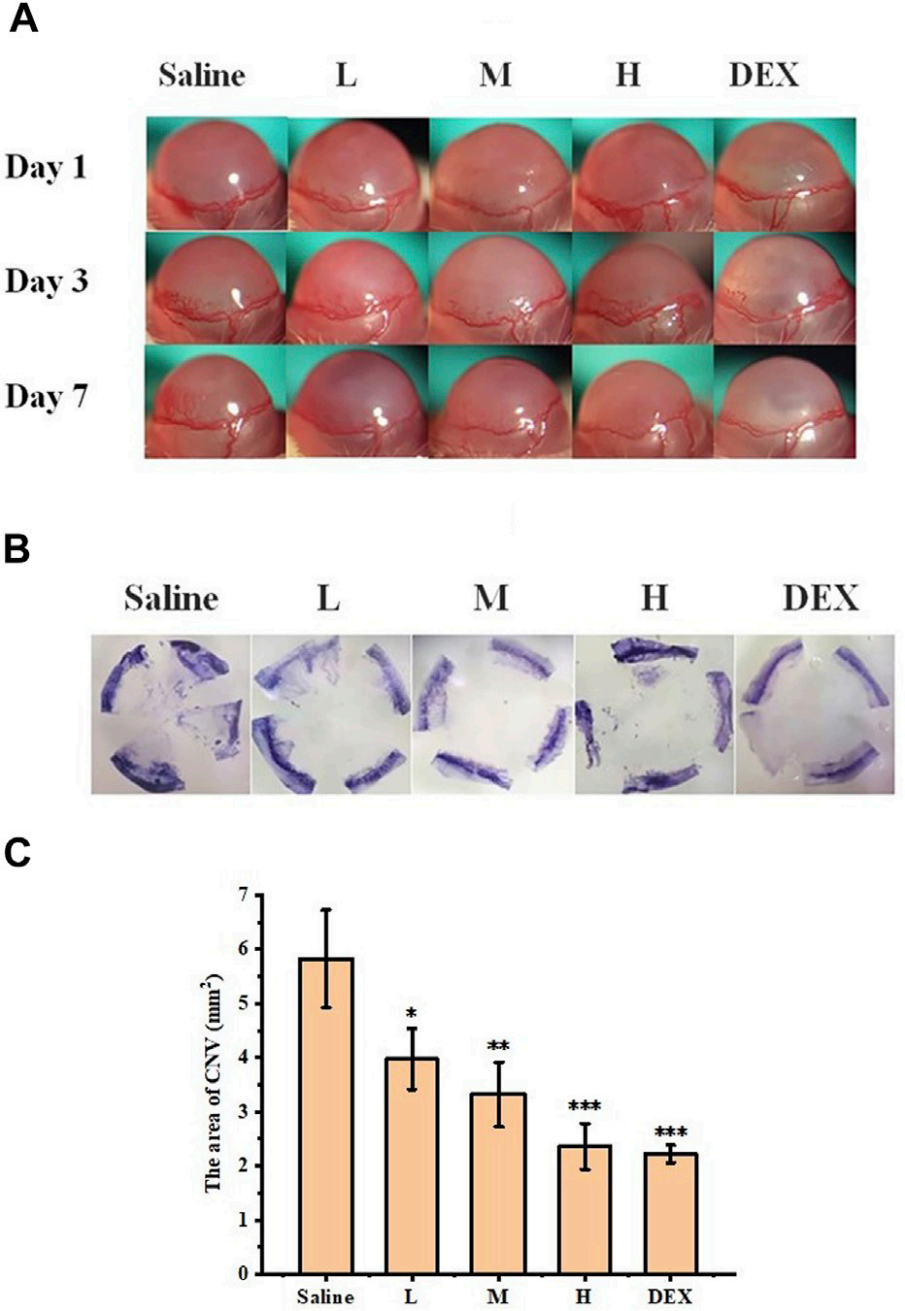
Inhibitory effect of STB-ME on CNV. **(A)** Slit lamp images of CNV on days 1, 3, and 7. **(B)** Hematoxylin staining images of different groups on day 7. **(C)** The area of CNV after modeling on day 7. (L: 0.025% STB-ME, M: 0.05% STB-ME, H: 0.1% STB-ME; *p < 0.05, **p < 0.001, compared to saline; mean ± SD, *n* = 3).

The inhibitory effect of STB-ME on CNV was confirmed in a mouse model of alkali burn-induced CNV. The burning area and corneal defect in the fluorescein sodium images (Figure 9) after modeling on day 0 were consistent, indicating that the models of each group were consistent. CNV images taken by slit lamp (Figure 10A) were used to observe the growth of vessels in different groups on days 1, 3 and 7, which showed that the growth trend of vessels in the saline group was the fastest. The results of the H&E staining images were performed in Figure 10B. In addition, the CNV area chart (Figure 10C) on day 7 after alkali burns showed that the CNV areas of the 0.025% (3.97 ± 0.56 mm^2^), 0.05% (3.32 ± 0.60 mm^2^) and 0.1% STB-ME groups (2.36 ± 0.42 mm^2^) were significantly lower than that in the saline group (5.83 ± 0.90 mm^2^) (*p* < 0.05), and there was no significant difference between the 0.1% STB-ME and DEX groups (2.22 ± 0.16 mm^2^) (*p* > 0.05).

#### 3.7.2 Histopathological examination

**FIGURE 11:**
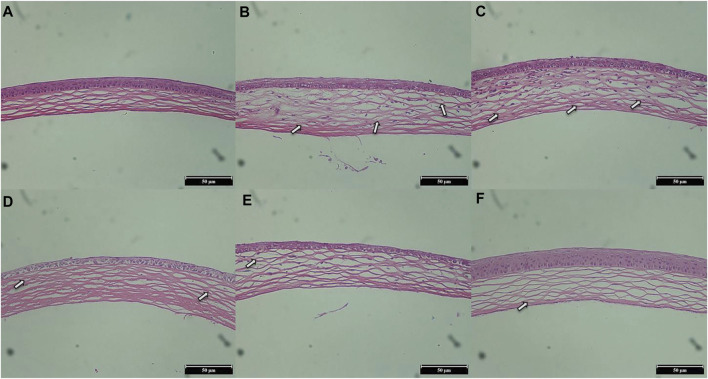
H&E staining images of corneas on day 7 (×200). **(A)** The normal group, **(B)** the saline group, **(C)** the 0.025% STB-ME group, **(D)** the 0.05% STB-ME group, **(E)** the 0.1% STB-ME group and **(F)** the DEX group.

H&E staining was used to evaluate the structural integrity and morphology of corneas in different groups of mice. As shown in Figure 11, the cornea of the normal group (Figure 11A) was neat and intact, with a uniform arrangement of collagen fibers in the stroma and no damage to the epithelial cells. The corneal image of the saline group (Figure 11B) showed obvious angiogenesis and disordered arrangement of stromal collagen fibers, and the arrangement of epithelial cells was irregular. Compared with the saline group, the blood vessels were decreased and the epithelial cells were intact in the L group (Figure 11C) and the M group (Figure 11D), indicating that their corneal histopathology was improved to a certain extent. In addition, the condition of the H group (Figure 11E) was similar to that of the DEX group (Figure 11F): the blood vessels were significantly reduced, the matrix collagen fibers were neatly arranged, and the corneal tissues were in good condition. These H&E analysis results were consistent with the CNV area results, indicating that STB-ME was reliable in inhibiting CNV.

#### 3.7.3 Enzyme-linked immunosorbent assay (ELISA)

**FIGURE 12:**
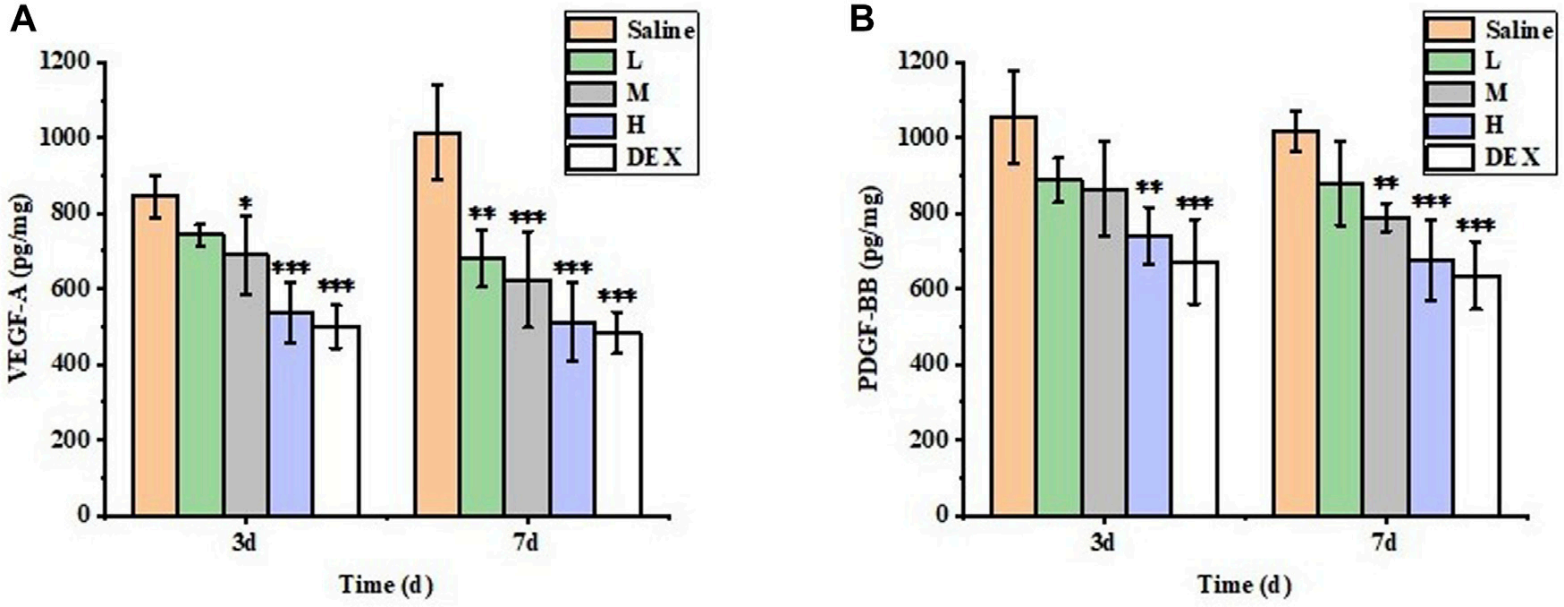
The protein expression levels of **(A)** VEGF-A and **(B)** PDGF-BB in the cornea were detected by ELISA. (L: 0.025% STB-ME, M: 0.05% STB-ME, H: 0.1% STB-ME; *p < 0.05, **p < 0.01, ***p < 0.001, compared to saline; mean ± SD, *n* = 5).

The inhibitory effect of each group on CNV was evaluated by measuring the contents of VEGF-A and PDGF-BB in total protein in different groups by ELISA (Figure 12). On day 7, the protein levels of VEGF-A and PDGF-BB in the M, H and DEX groups were significantly lower than those in the saline group (*p* < 0.05), and there was no significant difference in the levels of these factors between the H and DEX groups (*p* > 0.05). The results showed that STB-ME inhibited the expression of the protein factors VEGF-A and PDGF-BB to reduce the growth of CNV.

## 4 Discussion

CNV is a common ocular disease that can lead to blindness in severe cases. Reportedly, STB could reduce the growth of CNV by inhibiting VEGF-A and PDGF-BB factors, but the clinical application of STB in ophthalmology has been restricted due to its poor water solubility (Sedighi et al., 2020). However, an ME carrier could improve the solubility of STB if an oil phase with high solubility was selected, and surfactants and cosurfactants could be added to reduce tension and promote miscibility (Callender et al., 2017). In this study, an STB-ME was developed and successfully demonstrated to be applicable to CNV by *in vitro* and *in vivo* studies.

ME is commonly considered a thermodynamically stable system and has an optically transparent appearance; although theoretically it is spontaneously formed, a small energy input could help overcome kinetics barriers and mass transportation (Gonzalez-Fernandez et al., 2021). In our investigation, gentle agitation with a magnetic stirrer was used in the preparation process for STB-ME, and a short-term storage test indicated good physical stability.

Moreover, the mucoadhesive properties and small size of nanocarriers could enhance ocular bioavailability by increasing their residence in the cul-de-sac and promoting uptake into corneal or conjunctival tissues through endocytotic mechanisms (Souto et al., 2010). SH is a commercially available form of hyaluronic acid (HA), which is composed of repeating units of D-glucuronic acid and N-acetyl-D-glucosamine units, and has attracted significant interest due to its enhanced intraocular permeation, longer retention times, high physiological stability, inherent biocompatibility, and biodegradability. SH exhibits good mucoadhesive capabilities, readily forming hydrogen bonds with glycoproteins within the aqueous layer of tears and acting as a protective coating on corneal epithelial cells. Such binding forces significantly increase the precorneal residence time of SH-based formulations (Casey-Power et al., 2022). The viscosity of carriers also plays an important role in enhancing the corneal penetration (Awwad et al., 2017). In our study, SH was added to the STB-ME, and the results demonstrated that the drug levels in the cornea and conjunctiva were significantly higher in the STB-ME with SH group than in the group without SH. The pharmacokinetic results showed strong evidence that STB-ME increased the bioavailability of STB in the cornea and conjunctiva, because the addition of SH could prolong the retention time of STB in the ocular surface, improve the absorption time and reduce its clearance rate. A previous study showed that the use of 0.25% SH as a carrier increased the retention time of gentamicin in the cornea for at least 10 min and improved corneal bioavailability by topical administration (Bernatchez et al., 1993). In addition, it has been proven that 0.125% SH can increase the drug concentration of h-maucarpine hydrochloride solution in the cornea and aqueous humor (2-fold) (Camber and Edman, 1989). These experimental results were consistent with ours. On the other hand, it has been reported that the penetration of nanoparticles across the cornea is size-dependent, and approximately 40 nm or less of nanoparticles can diffuse across the stroma after topical application (Attama et al., 2009; Mohammadpour et al., 2014; Rajapakshal et al., 2015). The size of STB-ME prepared by optimization with CCD-RSM in our investigation was approximately 19 nm, and the PDI was small, which was beneficial for helping the drug penetrate across the ocular tissues after topical application.

MEs for ocular drug delivery could achieve sustained drug release after topical application and establish a higher drug level by promoting its penetration into the deeper layers of cornea, thus reducing the frequency of administration and side effects of the drugs, thus increasing patient compliance and ocular bioavailability compared to conventional eye drops (Gawin-Mikolajewicz et al., 2021). In this investigation, the administration of STB-ME twice a day achieved an effect on alkali burn-induced CNV in mice. Some drugs, such as naringenin (Ma et al., 2022), prednisolone (Hamed et al., 2022), triamcinolone acetonide (Mahran et al., 2021), brimonidine tartrate (Gautam and Kesavan, 2021) and cyclosporine (Coursey et al., 2018), have been loaded into ME systems for the treatment of ocular disorders.

According to the published literature (Ma et al., 2022), and it has also reported that at least 95% of the dose administered is eliminated systemically via the conjunctiva and nasolacrimal duct (Bachu et al., 2018), and a human tear has a total volume of approximately 7–30 μL with a turnover rate of 0.5–2.2 μL/min, and tear film has a rapid restoration time of 2–3 min (Janagam et al., 2017). Therefore, the toxicity of human corneal epithelial cells was evaluated at 50- and 100-fold dilutions of samples, i.e., 20 and 10 μg/mL, respectively. The HCEC cytotoxicity was positively correlated with drug concentration; thus, it was important to choose the right concentration. Although the toxicity at 10 μg/mL STB began to appear at 4 h, the concentration of the drug in rabbit corneas could not be reached and maintained at 10 μg/mL for such a long time; thus, the toxicity of the drug needs to be further studied. Eye safety has always been a major concern in the study of ophthalmic drug administration, and rabbits were selected for study because they are more sensitive to eye irritation than humans. In this study, the 0 score and complete section showed that STB-ME did not cause damage to the eye in the ocular irritation test.

The CNV area of 0.05% and 0.1% STB-ME was significantly reduced on day 7 compared with that of saline. In addition, the levels of VEGF-A and PDGF-BB in the experimental group were significantly reduced, indicating that STB inhibits the growth of CNV through at least two pathways, and previous studies have shown that the effect of inhibiting both factors is significantly different than that of only acting on VEGF-A (Lu et al., 2021). Reportedly, DEX is not suitable for clinical use because it often causes adverse reactions, such as glaucoma and cataracts, by either topical and systemic administration (Maeng et al., 2019; Gaballa et al., 2021). Fortunately, the efficacy of 0.1% STB-ME in inhibiting CNV is similar to that of DEX, which is expected to be a potential alternative to DEX in the future for the treatment of CNV. In previous studies, the inhibitory effect of 0.5 mg/mL STB on VEGF was approximately 3-fold greater than that of 5 mg/mL bevacizumab by topical administration, and the inhibitory effect of 4 mg/mL bevacizumab on VEGF was more potent than that of 0.1% DEX by topical application (Perez-Santonja et al., 2010; Luis de Redin et al., 2019). However, in this study, the ability of 1 mg/mL STB-ME to inhibit CNV was similar to that of 0.025% DEX, but not stronger. It was reported that the alkali burn model involves a mixture of inflammation and VEGF overexpression (Amano et al., 1998; Yu et al., 2023), and the similar therapeutic effect of STB-ME and DEX on CNV in our investigation may be attributed to the inhibitory effect of DEX on inflammation to some extent. Therefore, whether anti-VEGF-overexpression or anti-inflammation plays a more critical role in the treatment of CNV needs to be further studied. In addition, we did not observe an inhibitory effect of SH on CNV (Supplementary Section S3).

## 5 Conclusion

In this investigation, STB-ME was successfully prepared, which greatly improved the solubility of STB, and its various characteristics were suitable for ocular application. STB-ME could be stored at room temperature and low temperature, and the formulation showed no ocular irritation effect in rabbits. In the pharmacokinetic study, the addition of SH to STB-ME significantly increased the bioavailability of STB in the cornea. In pharmacodynamics, 0.1% STB-ME had a similar effect to DEX in inhibiting CNV. Therefore, STB-ME is a potential ocular drug delivery system for the treatment of CNV.

## Data Availability

The raw data supporting the conclusion of this article will be made available by the authors, without undue reservation.
