# Supplementary material for: Preparation of a Sunitinib loaded microemulsion for ocular delivery and evaluation for the treatment of corneal neovascularization in vitro and in vivo
Source: Front Pharmacol. 2023 Jul 11;14:1157084. doi: 10.3389/fphar.2023.1157084 (PMC10366539; doi:10.3389/fphar.2023.1157084)
Supplement: Supplementary file 1 [file DataSheet1.PDF]

Supplementary Material

Preparation of a Sunitinib Loaded Microemulsion for Ocular Delivery and Evaluation for the Treatment of Corneal Neovascularization *in Vitro* and *in Vivo*

Jieran Shi[1], Jingjing Yang[2], Haohang Xu[1], Qing Luo[1], Jun Sun[1], Yali Zhang[3], Zhen Liang[2], Ningmin Zhao[1*] and Junjie Zhang[2*]

*** Correspondence:** Ningmin Zhao: znm188@188.com; Junjie Zhang: zhangjunjie@zzu.edu.cn

Section 1 Formulation Optimization

Materials

Lauroglycol 90 (LG90) and Lauroglycol FCC (LGFCC) were obtained from Gattefossé (Saint-Priest, France). Cremophor®EL35 (EL35) was purchased from Meilun Biotechnology Co., Ltd. (Dalian, China). Ethyl oleate was acquired from J&K Scientific (Beijing, China). Tween 80 was obtained from Sichuan Jinshan Pharmaceutical Co., Ltd. (Sichuan, China). Glycerol was acquired from Zhejiang Suichang Huikang Pharmaceutical Co., Ltd. (Zhejiang, China). Polyethylene glycol 400 (PEG 400) and Triton X-100 were purchased from Beijing Solarbio Science & Technology Co., Ltd. (Beijing, China). Kolliphor®HS15 (HS15) was acquired from Beijing Fengli Jingqiu Pharmaceutical Co., Ltd. (Beijing, China). Kollisolv MCT 70 was obtained from Shanghai Yunhong Pharmaceutical Excipients Co., Ltd. (Shanghai, China). Propylene glycol dicaprylate (PGD) was acquired from Shandong Yongxin Zhonghe Biological Technology Co., Ltd. (Shandong, China).

1.1 Screening of Oils, Surfactants and Cosurfactants

1.1.1 The Assay of Solubility of STB in Oils, Surfactants and Cosurfactants

Excess STB was added to oils, surfactants and cosurfactants, each of which was 2 g in a vial with closed cover, respectively. The mixtures were shaken (100 rpm, 37 ℃) in an oven controlled oscillator (JBQ-ZD, Changzhou Putian Instrument Manufacturing Co., Ltd., Changzhou, China) for 48 h after thoroughly being stirred (37 ℃, 600 rpm) by a smart magnetic stirrer (ZNCL-BS180*180, Zhengzhou Ketai Laboratory Equipment Co., Ltd., Zhengzhou, China) (Gupta et al., 2019). After the sample was centrifuged (12000 rpm, 10 min) by a centrifuge (MiniSpin®plus, Eppendorf, Hamburg, Germany), the supernatant (0.1 g) was taken and diluted with methanol. The content of STB in the sample was detected by a UV detector (UV-1800SPC, Macy (China) Instruments Inc. Ltd., Shanghai, China) with a wavelength of 430 nm and each experiment was performed in triplicate to obtain reliable data.

It could be seen from Supplementary Figure S1 that the solubility of STB in TSP (37.16 ± 1.71 mg/g) is significant higher than that in PEG 400 (17.20 ± 0.46 mg/g), Glycerol (1.64 ± 0.02 mg/g) and Triton X-100 (14.17 ± 0.13 mg/g) ($P < 0.05$). In addition, the solubility of STB in OA (100.14 ± 1.48 mg/g) is significant higher than that in LG90 (16.73 ± 0.80 mg/g), LGFCC (12.85 ± 1.70 mg/g), ethyl

oleate (0.86 ± 0.04 mg/g), PGD (4.44 ± 0.15 mg/g) and Kollisolv MCT 70 (1.73 ± 0.04 mg/g) (P < 0.05). Therefore, TSP and OA were selected as cosurfactant and oil phase. However, there was no significant difference in the solubility of STB in CRH 40 (16.45 ± 0.27 mg/g), EL35 (17.57 ± 0.34 mg/g), HS15 (15.63 ± 1.17 mg/g) and Tween 80 (13.51 ± 0.27 mg/g), thus their emulsifying ability for OA to be further tested to select the most suitable surfactant.

1.1.2 The Evaluation of Surfactants for Emulsification Ability

Surfactants (CRH 40, EL35, HS15 and Tween 80, 0.6 g/each) were accurately weighed and added to 0.3 g of OA, respectively. Then, the mixture was stirred (37 °C, 600 rpm) until thoroughly homogenized. Each mixture (0.05 g) was added in a 50 mL volumetric flask filled with purified water at the same temperature, respectively. After the volumetric flask was turned upside down 100 times, it was placed at 25 °C for 2 h. The transmittance (%) of the samples was measured at 650 nm by a UV- spectrophotometer, and the mean values were compared (Farghaly et al., 2018). In this experiment, each sample was made in triplicate.

The ability of surfactants to emulsify OA was regarded as an important indicator for selection, which was indicated by comparison of transmittance. The transmittance of various surfactants was compared (Supplementary Figure S2), from which it could be seen that the transmittance of CRH 40 (90.12 ± 0.15 %) was the highest among these surfactants, including EL35 (79.03 ± 0.15 %), HS15 (77.27 ± 0.44 %) and Tween 80 (64.81 ± 1.78 %). CRH 40 has been widely used as an emulsifier for oil-in-water microemulsion drug delivery systems due to its excellent chemical stability (Lin et al., 2021). Therefore, CRH 40 was selected as the suitable surfactant for the following study.

1.2 Construction of Pseudo Ternary Phase Diagrams

The selected surfactant and cosurfactant were thoroughly mixed at mass ratios (Km) of 1:1, 2:1, 3:1, 4:1 and 5:1 by stirring with a smart magnetic stirrer (37 °C, 600 rpm). The selected oil according to the solubility was mixed with the mixtures of the surfactant and cosurfactant in a glass beaker, respectively, and their proportions were 1:9, 2:8, 3:7, 4:6, 5:5, 6:4, 7:3, 8:2, and 9:1, respectively (Hu et al., 2014). Then, purified water at the same temperature was dropwise added to the beaker and gently stirred until a clear and transparent liquid appeared. The solution made by the above-mentioned method was commonly named ME, and the quantity of the purified water added was weighed and recorded. Finally, the mass proportions of purified water, oil and mixed surfactants to the total were taken as coordinate axes, and pseudo ternary phase diagrams were plotted using Origin software (Version 2019b, OriginLab, Northampton, USA).

The pseudo ternary phase diagrams were established to obtain the proportion range of each component in the microemulsion region, and the influence of different Km on the microemulsion region areas were investigated. OA (oil phase), CRH 40 (surfactant) and TSP (cosurfactant) were determined as ME components by screening the solubility of oils and cosurfactants and comparing the emulsifying ability of surfactants. Pseudo ternary phase diagrams with different Km values (Km = 1, 2, 3, 4 and 5) are shown in Supplementary Figure S3, which shows that when Km from 4:1 to 5:1, the area of the microemulsion region was not significantly increased, therefore, the optimal range of Km values was 1 to 4 and oil was 0.2 to 0.6 g in the further studies.

1.3 Experimental Design

Expert Design software (Version 12, USA) was used to conduct two-factor and five-level central composite design (CCD) experiments to determine the optimal STB-ME formulation. In the design, the weight of oil (g) and Km (w/w) were independent variables and expressed as X_1 and X_2, respectively, and the droplet size (DS) and polydispersity index (PDI) were dependent variables and expressed as Y_1 and Y_2, respectively (Agrawal et al., 2021; Lin et al., 2021). After the samples were prepared according to 13 formulations generated by the software, curve fitting was performed to obtain the optimal formulation. The design of the independent variables is shown in Supplementary Table S1.

The CCD-RSM program of Design Expert software (Version 12, USA) was used to optimize composition ratio in the STB-ME formulation. Based on the two-factor and five-level design, 13 formulations are listed in Supplementary Table S2. The equations of oil (X_1), Km (X_2) and DS (Y_1) and PDI (Y_2) are obtained by mathematically fitting the model as follows:

$$\boldsymbol{Y_1 = 164.34 + 41.18X_1 - 12.62X_2 + 10.05X_1X_2 - 43.08{X_1}^2 + 3.67{X_2}^2 + 27.37{X_1}^2X_2 - 11.03X_1{X_2}^2\ (R^2 = 0.98)}$$

$$\boldsymbol{Y_2 = 0.2128 - 0.0187X_1 + 0.0067X_2 - 0.0110X_1X_2 + 0.0175{X_1}^2 + 0.0475{X_2}^2 + 0.0068{X_1}^2X_2 - 0.0018X_1{X_2}^2\ (R^2 = 0.98)}$$

It could be seen from Supplementary Table S2 that the DS ranges from 12.32 nm to 187.50 nm, and the PDI ranges from 0.209 to 0.320. In addition, consider the penetration into ocular tissues of STB-ME and its uniformity, both the DS and PDI should be minimum. With the minimum DS and PDI as the desirability constraints, the formulation was optimized by the prediction function of Design Expert 12 software, and the composition of the optimal STB-ME was generated. The three-dimensional (3D) surface diagrams of the dependent variables DS (Y_1) and PDI (Y_2) are shown (Supplementary Figure S4). The predicted and measured values of droplet size and PDI are shown in Supplementary Figure S5 with good fitting.

Tables

Supplementary Table S1. Code and value of the variables in CCD-RSM for STB-ME.

**Factor (independent variables)**	**Level**				
	-1.414	-1	0	1	1.414
X_1 (oil, g)	0.2	0.2586	0.4	0.5414	0.6
X_2 (Km, w/w)	1	1.44	2.5	3.56	4
Responses (dependent variables)	Desirability constraints				
Y_1 (DS, nm)	Minimize				
Y_2 (PDI)	Minimize				

Abbreviations: DS, droplet size; PDI, polydispersity index.

Supplementary Table S2. Composition and response values of 13 STB-ME formulas (X_1: oil, X_2: Km, Y_1: Droplet size, Y_2: PDI).

	Factor		Response	
Run	X_1 (g)	X_2 (w/w)	Y_1 (nm)	Y_2
1	0.4	4.00	146.20	0.320
2	0.5414	1.44	137.90	0.252
3	0.4	2.50	165.50	0.212
4	0.5414	3.56	187.50	0.257
5	0.6	2.50	128.80	0.224
6	0.4	2.50	162.80	0.225
7	0.4	2.50	166.40	0.204
8	0.2586	1.44	97.71	0.271
9	0.4	1.00	181.90	0.301
10	0.2	2.50	12.32	0.277
11	0.4	2.50	160.00	0.214
12	0.4	2.50	167.00	0.209
13	0.2586	3.56	107.10	0.320

Figures

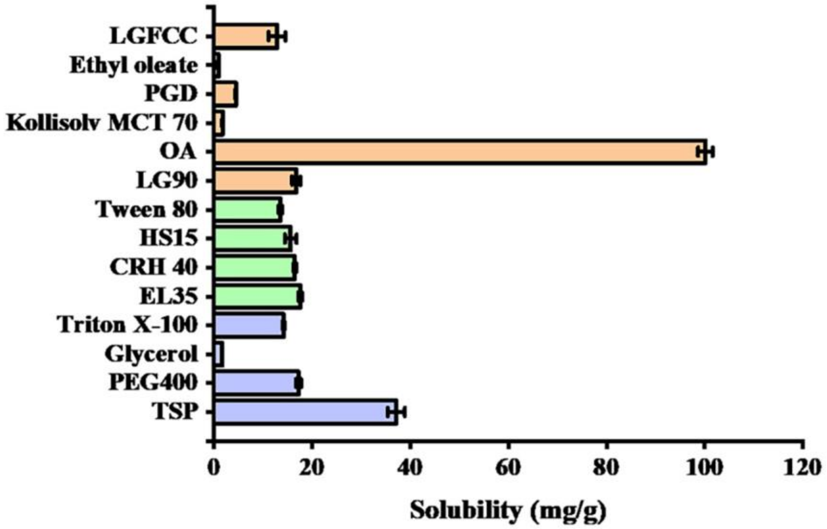

Supplementary Figure S1. Solubility values of STB in oils, surfactants and cosurfactants (mean ± SD, n = 3).

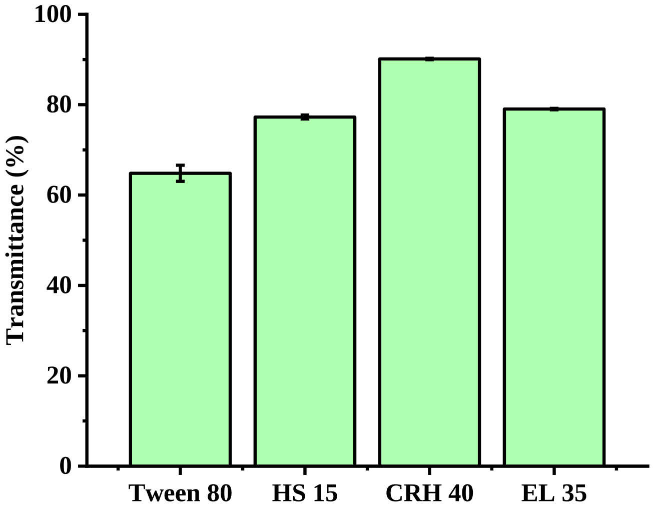

Supplementary Figure S2. Screening of emulsifying ability of surfactants (mean ± SD, n = 3).

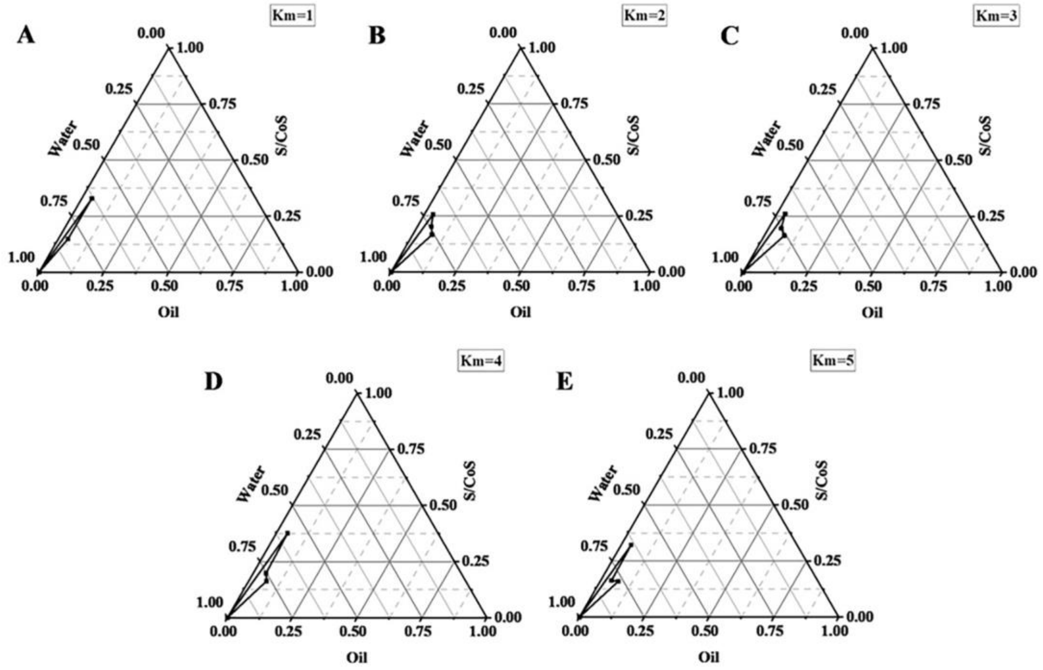

Supplementary Figure S3. Pseudo ternary phase diagrams prepared with different Km (w/w). **(A)** Km = 1:1, **(B)** Km = 2:1, **(C)** Km = 3:1, **(D)** Km = 4:1, (E) Km = 5:1.

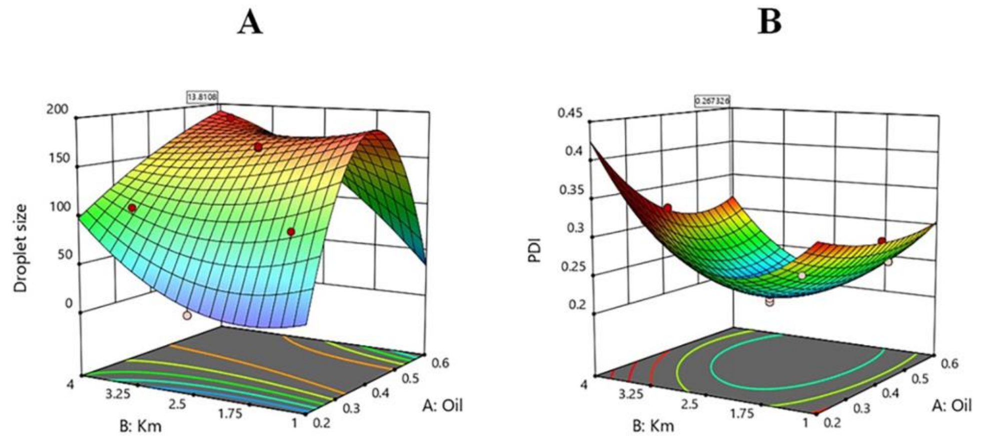

Supplementary Figure S4. 3D surface diagram of independent variables (oil and Km) and dependent variables (DS and PDI). **(A)** 3D Surface diagram of independent variables (oil and Km) and dependent variable (DS); **(B)** 3D Surface diagram of independent variables (oil and Km) and dependent variable (PDI).

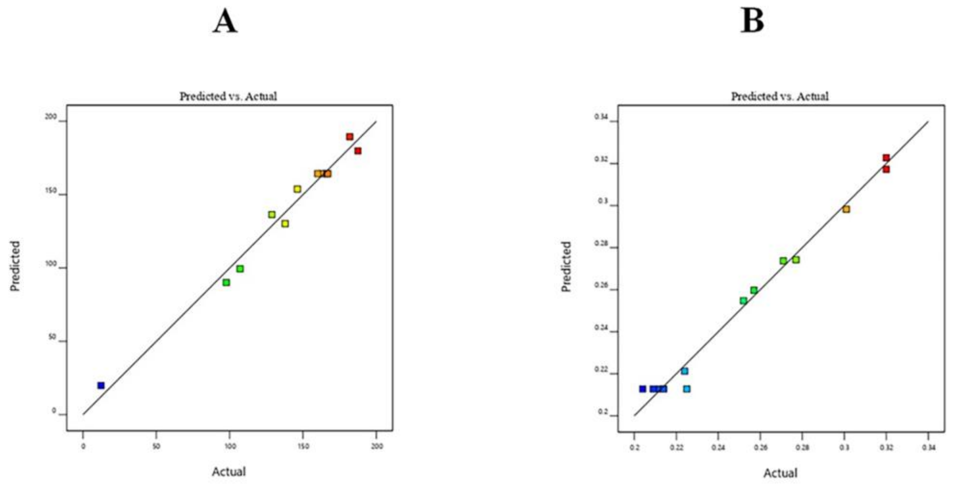

Supplementary Figure S5. The predicted and actual (measured) values of **(A)** droplet size and **(B)** PDI.

Section 2 Method validation parameters for the bioanalytical methods

The method validation parameters for the bioanalytical methods for quantification of STB in cornea and conjunctiva. The method was validated for specificity, linearity, recovery, precision and accuracy and stability.

2.1 Preparation of stock and standard solutions

STB stock solution was prepared by exactly weighing 20 mg of compound in volumetric flask and completing the volume with methanol up to 20 mL, so that a 1 mg/mL solution was obtained. The stock solution was diluted with methanol to produce working standard solutions at concentrations of

20, 10, 3.33, 1.66, 0.55, 0.28 and 0.14 μg/mL for preparing cornea calibration standards and at concentrations of 8, 4, 1.33, 0.66, 0.22 and 0.11 μg/mL for preparing conjunctiva calibration standards. The STB working standard solutions (100 μL) were pipetted into centrifuge tubes and dried by nitrogen flow at room temperature. The pooled methanol extracting solution of blank cornea or conjunctiva were prepared by adding 10 times volume of methanol (v/w) to blank cornea or conjunctiva, sealed and stored at 4 ± 2°C for 24 hours and then the extracting solutions were used to prepare the calibration standards, quality control samples and other spiked samples. Calibration standards were prepared by adding a 400 μL aliquot of pooled methanol extracting solution of blank cornea or conjunctiva, i.e., solutions of cornea (5, 2.5, 0.83, 0.416, 0.139, 0.069 and 0.035 μg/mL) and conjunctiva (2, 1, 0.33, 0.165, 0.055 and 0.028 μg/mL). Subsequently, the tubes were vortexed for 30 s and centrifuged for 10 min at 12000 rpm (MiniSpin® Plus, Eppendorf, Germany) for analysis. Quality control samples of cornea (2.5, 0.416 and 0.069 μg/mL) and conjunctiva (1, 0.165 and 0.055 μg/mL) were prepared in a similar manner with appropriate working standard solution.

2.2 Specificity

The specificity was investigated by analyzing extracting solution of blank cornea and conjunctiva from six individual rabbits. Specificity was established by the lack of interfering peaks at the retention time for the STB. Typical chromatograms of blank cornea and conjunctiva sample, the standard solutions evaporated directly and reconstituted in methanol, the spiked pooled methanol extracting solution of blank cornea or conjunctiva and pharmacokinetic sample were shown in cornea (Supplementary Figure S6) and conjunctiva (Supplementary Figure S7).

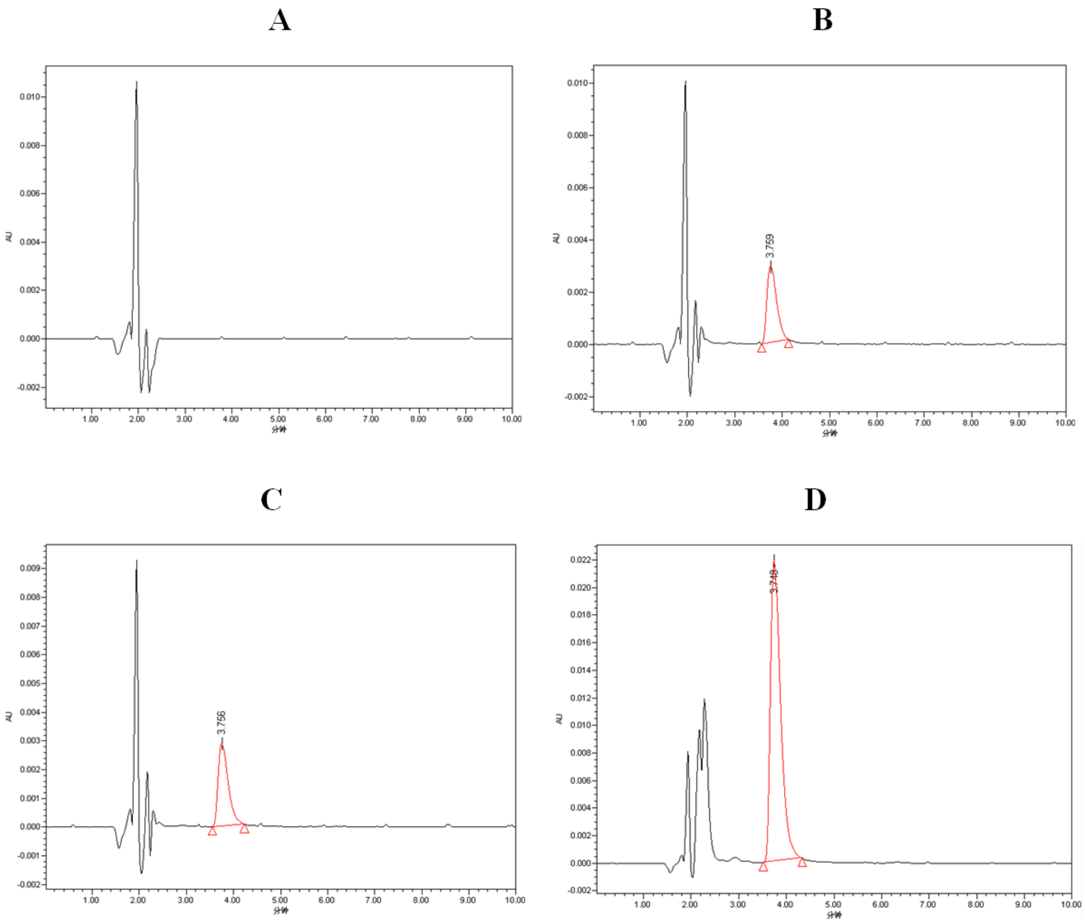

Supplementary Figure S6. Typical chromatograms of **(A)** methanol extracting solution of blank cornea, **(B)** the standard solutions evaporated directly and reconstituted in methanol, **(C)** spiked pooled methanol extracting solution of blank cornea and **(D)** pharmacokinetic sample at 15 min.

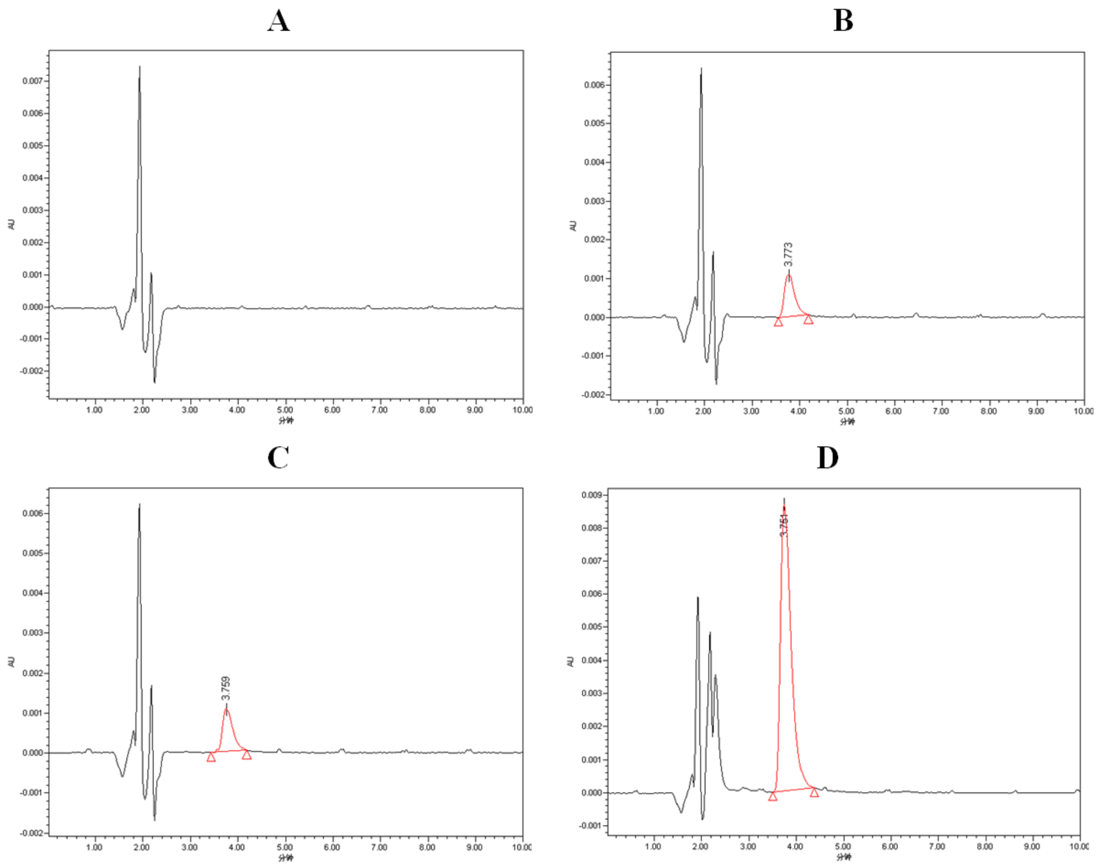

Supplementary Figure S7. Typical chromatograms of **(A)** methanol extracting solution of blank conjunctiva, **(B)** the standard solutions evaporated directly and reconstituted in methanol, **(C)** spiked pooled methanol extracting solution of blank conjunctiva and **(D)** pharmacokinetic sample at 15 min.

2.3 Linearity

The calibration curve was tested at different concentrations, covering a range of cornea (5, 2.5, 0.83, 0.416, 0.139, 0.069 and 0.035 μg/mL) and conjunctiva (2, 1, 0.33, 0.165, 0.055 and 0.028 μg/mL). The calibration curves were established by plotting the peak area versus concentration, and the obtained data were subjected to regression analysis using the ordinary least squares method. The linearity was evaluated by means of squared coefficient of determination (R^2). The limit of quantitation (LOQ) of the assay was assessed as the lowest concentration of the calibration curve that can be quantitatively determined within 20% accuracy and precision.

The linear equations of cornea were "y=101360x+130.28" on day 1, "y=111023x–953.66" on day 2 and "y=106981x–862.61" on day 3, where "y" is peak area and "x" is concentration of STB. The correlation coefficients (R^2) of the calibration curves for spiked cornea samples were 0.9999, 0.9999 and 0.9999 respectively, as could be seen in Supplementary Figure S8. And the LOQ value was 0.035 μg/mL.

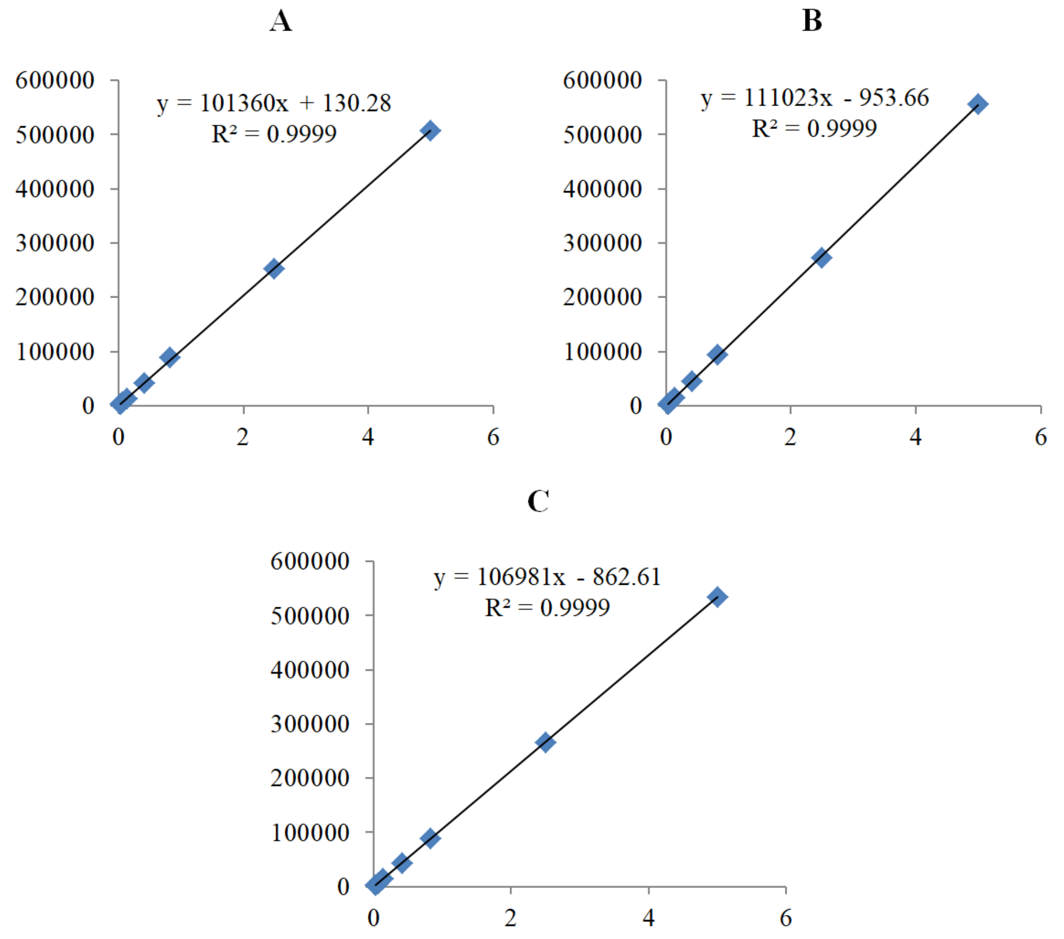

Supplementary Figure S8. Calibration curves of STB in cornea of day 1 **(A)**, day 2 **(B)** and day 3 **(C)**.

The linear equations of conjunctiva were "y=100619x–620.18" on day 1, "y=104256x–238.75" on day 2 and "y = 111032x-278.38" on day 3, where "y" is peak area and "x" is concentration of STB. The correlation coefficients (R^2) of the calibration curves for spiked conjunctiva samples were 0.9998, 0.9992 and 0.9999 respectively, as could be seen in Supplementary Figure S9. And the LOQ value was 0.028 µg/mL.

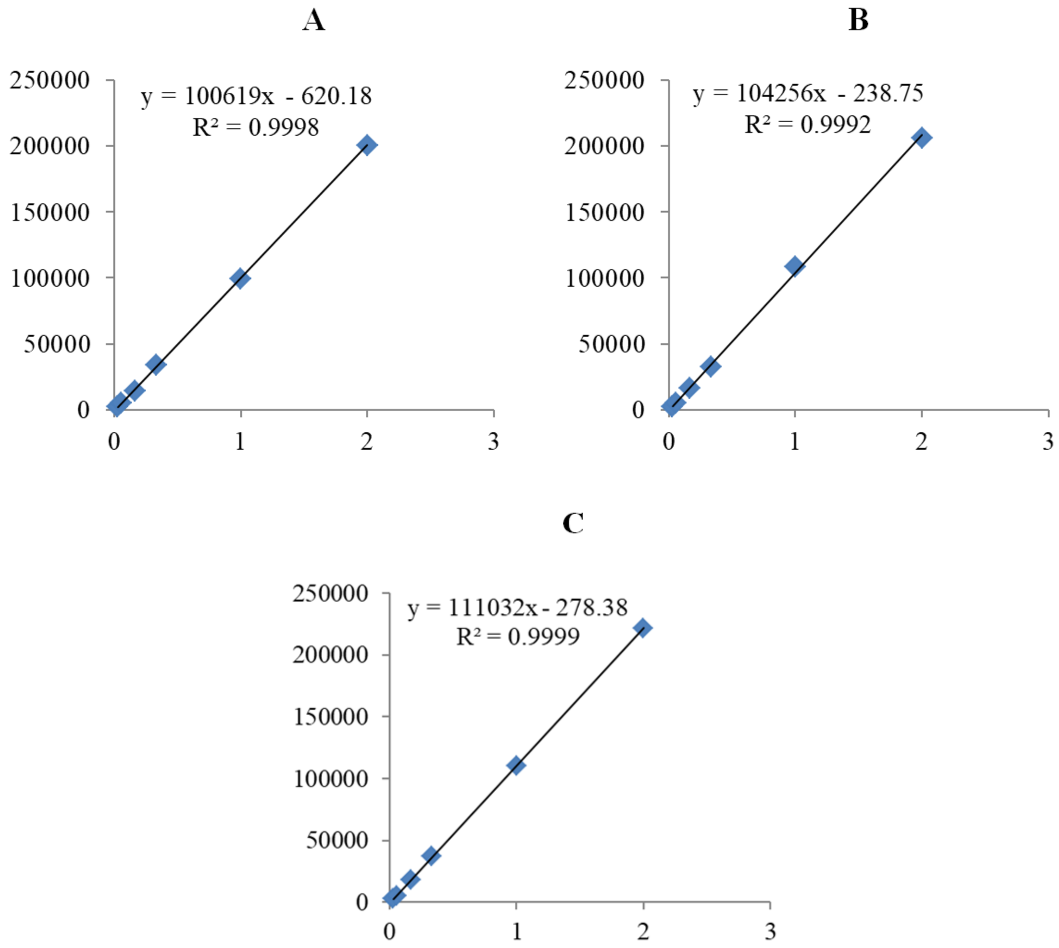

Supplementary Figure S9. Calibration curves of STB in conjunctiva of day 1 **(A)**, day 2 **(B)** and day 3 **(C)**.

2.4 Precision and accuracy

Precision and accuracy of this analytical method were determined using LOQ sample (0.035 μg/mL or 0.028 μg/mL) and quality control samples in five replicates of STB in pooled blank extracting solution for three consecutive days. The intra- and inter-batch precisions were calculated according to the relative standard deviation (% RSD) and accuracy was calculated according to the recovery value (% Re). The precision determined at LOQ should be within ±20%, while at other three concentration levels should be within ±15%. The accuracy determined at LOQ should be within 80 - 120%, while at other three concentration levels should be within 85 - 115%. Precision and accuracy data for intra-batch and inter-batch spiked cornea and conjunctiva samples were presented in Supplementary Table S3 and Supplementary Table S4.

Supplementary Table S3. Intra-batch assay precision and accuracy for STB (n=5).

Concentration of STB (μg/mL)		1st day		2nd day		3rd day	
		RSD (%)	Re (%)	RSD (%)	Re (%)	RSD (%)	Re (%)
conjunctiva	0.028	4.15	100.26±4.16	1.86	96.94±1.81	2.38	95.32±2.27
	0.055	2.34	97.34±2.28	1.85	92.03±1.70	0.72	92.21±0.66
	0.165	3.43	99.89±3.43	3.25	94.22±3.07	2.37	91.71±2.18
	1	4.09	106.36±4.35	3.17	105.41±3.34	4.04	107.49±4.34
cornea	0.035	2.25	99.81±2.25	1.174	108.49±1.27	2.79	107.92±3.01
	0.069	1.12	91.70±1.03	0.713	96.93±0.69	1.19	100.44±1.19
	0.416	1.97	101.88±2.01	1.660	101.31±1.68	1.69	104.97±1.78
	2.5	1.27	105.96±1.34	1.281	105.50±1.35	1.32	102.61±1.35

Supplementary Table S4. Inter-batch assay precision and accuracy for STB (n=15).

Concentration of STB (μg/mL)		RSD (%)	Re (%)
conjunctiva	0.028	2.59	97.51±3.45
	0.055	3.21	93.86±2.99
	0.165	4.40	95.27±4.46
	1	0.98	106.42±3.84
cornea	0.035	4.60	105.41±4.62
	0.069	4.56	96.35±3.83
	0.416	1.92	102.72±2.37
	2.5	1.73	104.69±1.98

2.5 Recovery

The extracting recovery was estimated by comparing the corresponding peak areas of the spiked pooled blank extracting solution of cornea and conjunctiva to those of the standard solutions evaporated directly and reconstituted in methanol. These experiments were performed in five replicates at three concentration levels for STB. The recoveries of STB from the spiked cornea samples at concentrations of 2.5, 0.416, 0.069 μg/mL were 100.07 ±0.35%, 104.63 ±1.81% and 100.37 ±0.80%, respectively (n = 3). The recoveries of STB from the spiked conjunctiva samples at concentrations of 1, 0.165, 0.055 μg/mL were 101.53 ±3.99%, 100.49 ±1.26% and 100.81 ±1.19%, respectively (n = 3).

2.6 Stability

The long-term stability of the STB in rabbit cornea and conjunctiva were evaluated by adding a 10 mg of blank cornea and conjunctiva to the spiked tube and storing at -80°C for 4 weeks. Autosampler stability was evaluated by transferring processed quality control samples of cornea and conjunctiva and maintaining in the autosampler rack at 25 ±2°C for 7 h at the same concentration levels as the

recovery experiment. The quality control samples were used for the bench-top stability study, which was performed at ambient temperature for 1 h, after which they were processed and analyzed. The stability of STB in rabbit cornea and conjunctiva after three freeze (-80℃) and thaw (room temperature) cycles were investigated by subjecting quality control samples to freeze and thaw cycles, each freeze cycle was at least for 12 h before it was thawed. All the stability samples were analyzed against a freshly prepared calibration curve. The stability data of STB were presented in Supplementary Table S5.

Supplementary Table S5. Stability of STB in the cornea and conjunctiva (n=5).

Concentration of STB (μg/mL)		Recovery of STB under following conditions (%, mean±SD)			
		Bench-top stability	Freeze/thaw stability	Long-term stability	Autosampler stability
conjunctiva	0.055	91.73±2.16	92.62±3.99	93.34±0.70	91.77±1.73
	0.165	90.72±3.26	94.03±2.52	96.99±3.38	95.34±3.02
	1	100.27±2.90	100.59±1.77	102.90±5.11	111.63±1.98
cornea	0.069	103.96±1.58	103.04±1.31	101.91±1.76	103.05±0.98
	0.416	109.24±1.55	103.41±1.74	105.29±2.52	100.26±1.47
	2.5	106.77±2.02	103.25±1.84	104.66±0.99	104.53±1.59

Section 3 Study on Anti-CNV of Sodium Hyaluronate in Mice

The method of anti-CNV study of sodium hyaluronate (SH) in mice was consistent with that in manuscript Section 2.11. The mice were divided into five groups (saline, blank-ME (contained SH), 0.1% STB-ME (without SH), 0.1% STB-ME and DEX), with 16 mice in each group. The experiment included observation and measurement of CNV, histopathological examination and enzyme-linked immunosorbent assay (ELISA).

3.1 Observation and Measurement of CNV

The burning area and corneal defect in the fluorescein sodium images after modeling on day 0 (Supplementary Figure S10) were consistent, indicating that the models of each group were consistent. The CNV images taken by the slit lamp (Supplementary Figure S11 A) were used to observe the growth of vessels in different groups on days 1, 3 and 7, which showed that the growth trend of vessels in the saline and blank-ME groups were the fastest. In addition, the CNV area (Supplementary Figure S11 C) on day 7 after alkali burns showed that the CNV area of the STB-ME (without SH) (2.88 ± 0.43 mm^2), STB-ME (2.37 ± 0.26 mm^2) and DEX groups (2.41 ± 0.13 mm^2) were significantly lower than that of the saline group (5.33 ± 0.81 mm^2) ($P < 0.05$), and there was no significant difference between the saline group and blank-ME group (5.30 ± 1.03 mm^2) ($P > 0.05$), which were similar to the results of the hematoxylin staining images (Supplementary Figure S11 B).

3.2 Histopathological Examination

H&E staining was used to evaluate the structural integrity and morphology of cornea in different groups of mice. As shown in Supplementary Figure S12, the cornea of the normal group (Supplementary Figure S12 A) was intact and neat, with a uniform arrangement of collagen fibers in the stroma and no damage to the epithelial cells. The corneal image of the saline group (Supplementary Figure S12 B) and the blank-ME group (Supplementary Figure S12 C) showed obvious angiogenesis and disordered arrangement of stromal collagen fibers, and the arrangement of

epithelial cells were irregular. In addition, the condition of STB-ME (without SH) group (Supplementary Figure S12 D) and the STB-ME group (Supplementary Figure S12 E) were similar to that of the DEX group (Supplementary Figure S12 F), their blood vessels were significantly reduced, the matrix collagen fibers were neatly arranged, and the corneal tissues were in good condition. These H&E analysis results were consistent with the CNV area results, indicating that SH did not play a role in inhibiting CNV.

3.3 Enzyme-Linked Immunosorbent Assay (ELISA)

The inhibitory effect of each group on CNV was evaluated by measuring the contents of VEGF-A and PDGF-BB in total protein in different groups by ELISA (Supplementary Figure S13). On days 3 and 7, the protein levels of VEGF-A and PDGF-BB in the STB-ME (without SH), STB-ME and DEX groups were significantly lower than those in the saline group ($P < 0.05$), and there was no significant difference in the levels of these factors between the saline group and blank-ME group ($P > 0.05$). It could be seen from the results that STB but not SH played an important role in the treatment of CNV by inhibiting the expression of the protein factors VEGF-A and PDGF-BB.

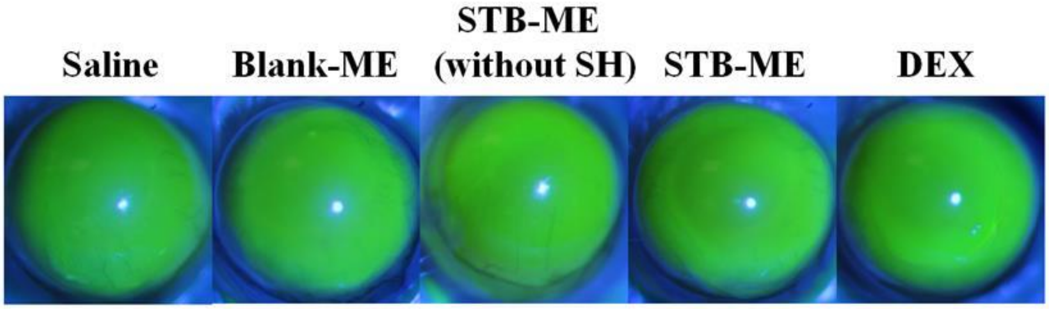

Supplementary Figure S10. The fluorescein sodium images after modeling on day 0.

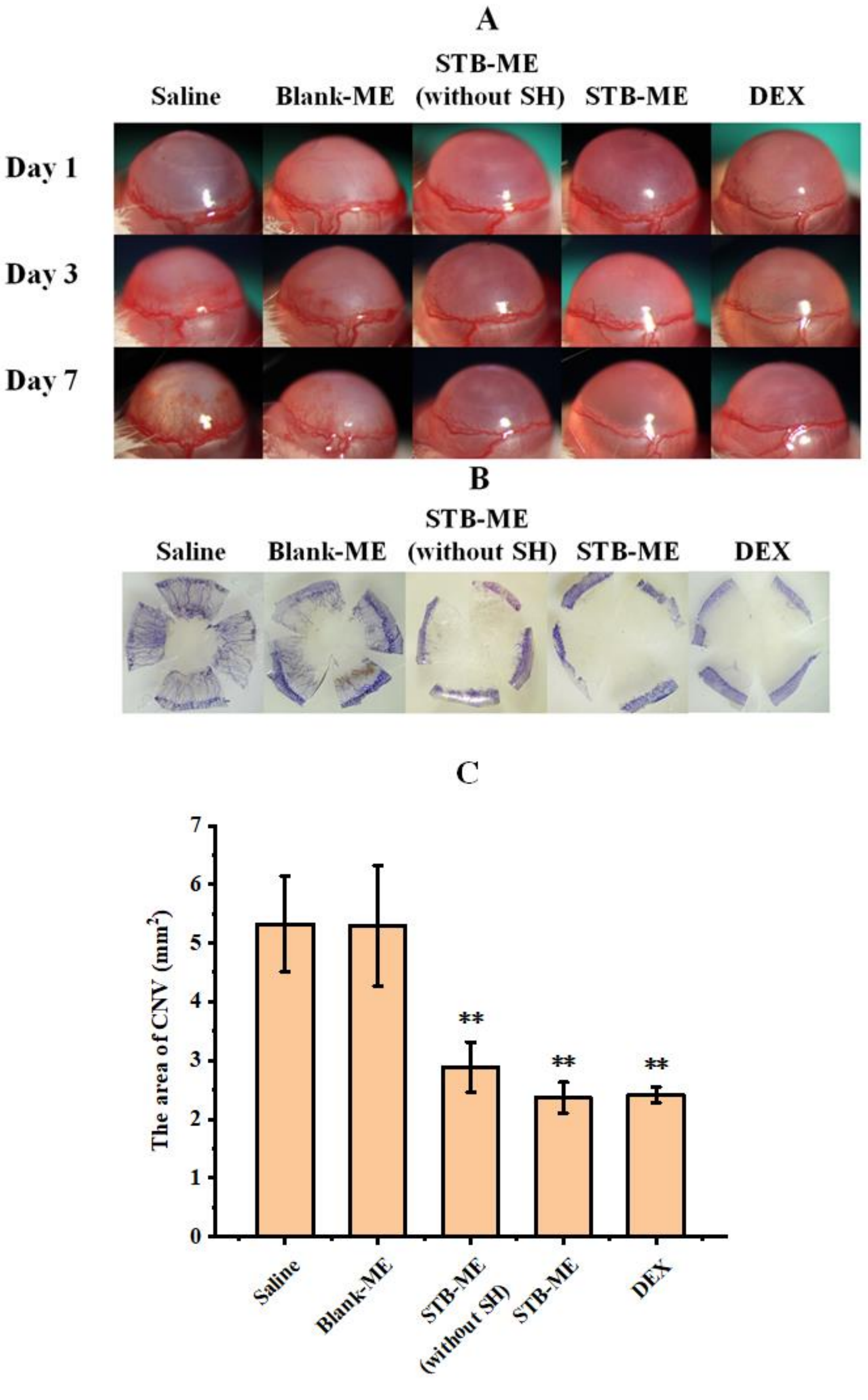
A
Saline
Blank-ME
STB-ME (without SH)
STB-ME
DEX
Day 1
Day 3
Day 7
B
Saline
Blank-ME
STB-ME (without SH)
STB-ME
DEX
C
The area of CNV (mm^2)
0
1
2
3
4
5
6
7
**
**
**
Saline
Blank-ME
STB-ME (without SH)
STB-ME
DEX

Supplementary Figure S11. Inhibitory effect of STB-ME on CNV. **(A)** Slit lamp images of CNV on days 1, 3 and 7. **(B)** The hematoxylin staining images of different groups on day 7. **(C)** The area of CNV after modeling on day 7. (**P* < 0.05, ***P* < 0.001, compared to Saline; mean ± SD, n = 3)

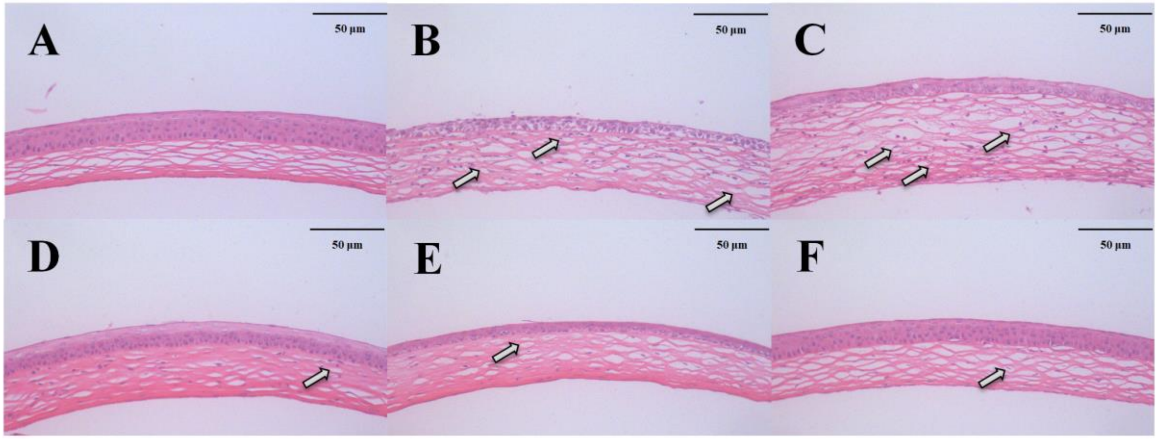

Supplementary Figure S12. H&E staining images of cornea on day 7 (×200). **(A)** the normal group, **(B)** the saline group, **(C)** the blank-ME group, **(D)** the STB-ME (without SH) group, (E) the STB-ME group, **(F)** the DEX group.

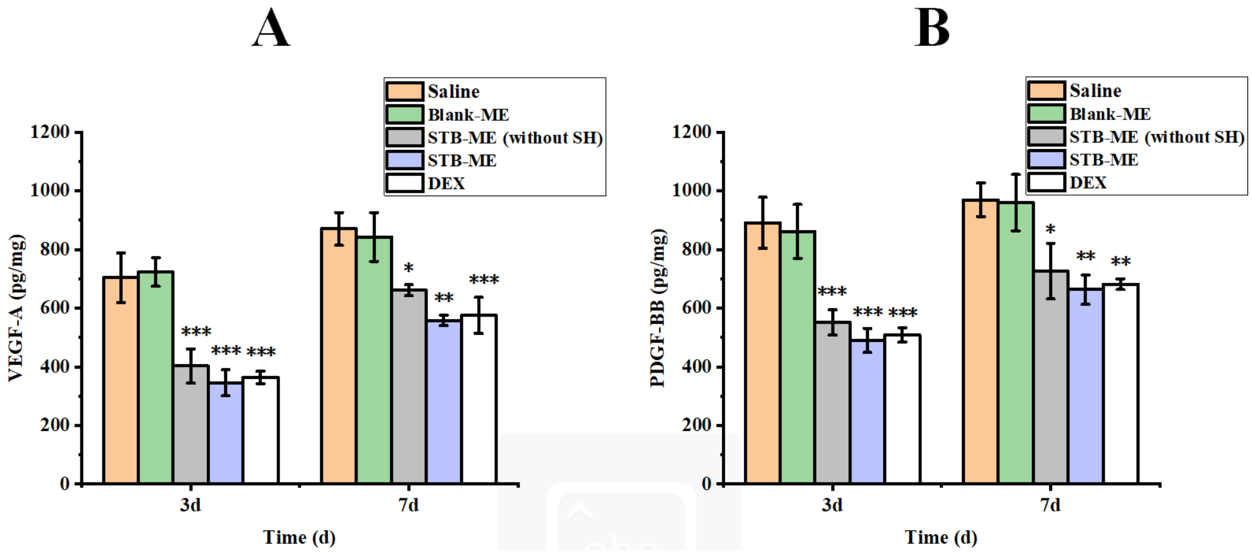

Supplementary Figure S13. The protein expression levels of VEGF-A and PDGF-BB in cornea detected by ELISA. (**P* < 0.05, ***P* < 0.01, ****P* < 0.001, compared to Saline; mean ± SD, n = 5).

Reference

Agrawal, V., Patel, R., Patel, M., Thanki, K. and Mishra, S. (2021). Design and evaluation of microemulsion-based efinaconazole formulations for targeted treatment of onychomycosis through transungual route: Ex vivo and nail clipping studies. *Colloids Surf B Biointerfaces*. 201, 111652. doi: 10.1016/j.colsurfb.2021.111652

Farghaly, D. A., Aboelwafa, A. A., Hamza, M. Y. and Mohamed, M. I. (2018). Microemulsion for topical delivery of fenoprofen calcium: in vitro and in vivo evaluation. *J Liposome Res*. 28, 126-136. doi: 10.1080/08982104.2017.1281951

Gupta, A., Nayak, K. and Misra, M. (2019). Cow ghee fortified ocular topical microemulsion; in vitro, ex vivo, and in vivo evaluation. *J Microencapsul*. 36, 603-621. doi: 10.1080/02652048.2019.1662121

Hu, L., Hu, Q. and Yang, J. (2014). Enhancement of transdermal delivery of ibuprofen using microemulsion vehicle. *Iran J Basic Med Sci*. 17, 760-766. doi:

Lin, L., Asghar, S., Huang, L., Hu, Z., Ping, Q., Chen, Z., et al. (2021). Preparation and evaluation of oral self-microemulsifying drug delivery system of Chlorophyll. *Drug Dev Ind Pharm*. 47, 857-866. doi: 10.1080/03639045.2021.1892746
